# Cold- and hot-classified botanical drugs differentially modulate gut microbiota: linking TCM emic classification to microbial ecology

**DOI:** 10.3389/fphar.2025.1545619

**Published:** 2025-05-16

**Authors:** Huan Yang, Chenyi Shao, Zhihao Liu, Xiaoyu Zhang, Yinhui Liu, Jing Xiao, Li Tang

**Affiliations:** ^1^ Department of Microecology, College of Basic Medical Sciences, Dalian Medical University, Dalian, China; ^2^ Department of Oral Pathology, College of Stomatology, Dalian Medical University, Dalian, China

**Keywords:** gut microbiota dysbiosis, traditional Chinese medicine, *Akkermansia*, *Clostridia*, emic classification, TCM property

## Abstract

**Introduction:**

Traditional Chinese Medicine (TCM) classifies botanical drugs based on their thermal properties (an emic classification system), categorizing them as “cold” (e.g., “clearing heat” for anti-inflammatory effects) or “hot” (e.g., “warming the middle” for metabolic enhancement). However, the specific roles of these botanical drugs in restoring gut microbiota dysbiosis remain unclear. This study aimed to explore whether TCM-classified cold and hot botanical drugs differentially restore gut microbiota dysbiosis and host physiology in antibiotic-treated mice.

**Methods:**

Mice with antibiotic-induced dysbiosis were treated with eight TCM-classified botanical drugs (four cold: *Rheum palmatum* L., *Scutellaria baicalensis* Georgi, *Senna alexandrina* Mill., *Coptis chinensis* Franch.; four hot: *Codonopsis pilosula* (Franch.) Nannf., *Astragalus membranaceus* (Fisch.) Bunge, *Angelica sinensis* (Oliv.) Diels, *Panax ginseng* C.A.Mey.) for 20 days. Gut microbiota were analyzed via 16S rRNA sequencing on days 5, 10, 15, and 20, alongside physiological parameters including blood glucose, serum lipids, TNF-α, adiponectin, and intestinal histomorphology.

**Results:**

By day 20, all botanical drugs restored the diversity and ranking of dominant genera (those with >10% abundance, such as *Lactobacillu*s and *unclassified Muribaculaceae*). However, cold-classified drugs, traditionally associated with anti-inflammatory effects, selectively enriched anti-inflammatory taxa, including *Akkermansia* and *Bifidobacterium*. In contrast, hot-classified drugs, linked to metabolic enhancement, promoted metabolic-modulating genera such as *Clostridia* and *Eubacterium coprostanoligenes*. These differential enrichments corresponded with the therapeutic principles defined by TCM: cold-classified drugs reduced serum TNF-α levels (*P* < 0.01), demonstrating anti-inflammatory effects, whereas hot-classified drugs improved lipid profiles (TG: *P* < 0.001), thereby promoting metabolic modulation.

**Discussion:**

TCM-classified cold and hot botanical drugs universally stabilize dominant microbiota while differentially modulating low-abundance taxa. The enrichment of *Akkermansia* (cold) and *Clostridia* (hot) offers a microbiota-driven validation of TCM’s empirical classification framework. These findings connect traditional knowledge with microbial ecology, underscoring the potential of TCM-guided microbiota modulation for precision therapies.

## Graphical Abstract

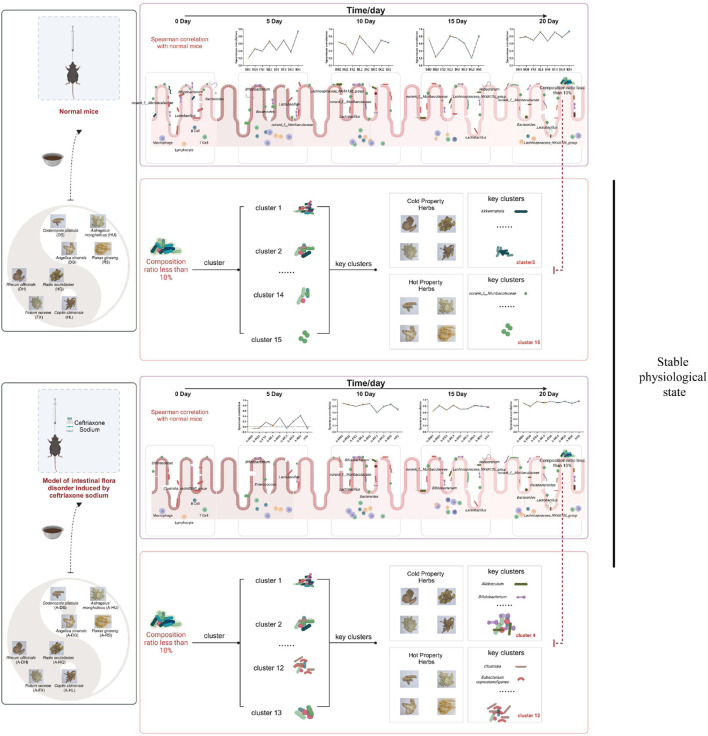

## 1 Introduction

The gut microbiota, recognized as a vital “organ” within the human body, plays a crucial role in maintaining homeostatic balance by regulating energy metabolism, immune responses, and material transformations (de Vos et al., 2022). Its diversity and stability are hallmarks of health, while dysbiosis is closely associated with disease pathogenesis (Sender et al., 2016; Fan and Pedersen, 2021). Broad-spectrum antibiotics, such as ceftriaxone—a third-generation cephalosporin—disrupt the composition of the gut microbiota by reducing diversity and increasing the prevalence of opportunistic pathogens (Yang et al., 2023). Restoring the balance of the microbiota following antibiotic exposure presents a significant therapeutic challenge, particularly for disorders linked to metabolic and immune dysregulation.

Botanical drugs, such as *Coptis chinensis* Franch. and *Codonopsis pilosula* (Franch.) Nannf., which are integral to TCM, are increasingly recognized for their effects on microbiota modulation (Weersma et al., 2020). In TCM theory, botanical drugs are empirically classified as cold (e.g., *Coptis chinensis* Franch.) or hot (e.g., *Panax ginseng* C.A.Mey.) based on their perceived therapeutic functions—termed “clearing heat” (anti-inflammatory) or “warming the middle” (metabolic enhancement). This classification system is rooted in centuries of observational practice and represents an emic (culture-specific) perspective, as defined by Headland et al. (1992), rather than relying on biochemical metrics (Xu et al., 2019). In TCM, the emic classification reflects culture-specific therapeutic observations, whereas an etic approach would focus on biochemical metrics. This study bridges these perspectives by investigating microbial biomarkers associated with TCM properties. Preliminary studies suggest tentative associations between these classifications and phytochemical composition; for instance, alkaloids (e.g., berberine) are enriched in cold-classified drugs, while saponins (e.g., ginsenosides) dominate hot-classified counterparts (Peng et al., 2023). However, such associations remain largely descriptive, and the mechanisms linking TCM classifications to microbiota modulation are not well understood.

To bridge this gap, we aimed to test two hypotheses:

1) Botanical drugs classified as cold or hot in TCM ifferentially restore dysbiotic gut microbiota by enriching bacterial taxa that align with their traditional therapeutic principles, such as anti-inflammatory effects versus metabolic modulation.
2) Taxon-specific changes in microbiota correlate with measurable host outcomes, such as reduced inflammation and improved lipid profiles.

## 2 Materials and methods

### 2.1 Reagents

*Rheum palmatum* L. (Rhei Radix et Rhizoma, origin: Sichuan Province, batch number 220221; taxonomically validated via MPNS and POWO; voucher specimen No. DMU-MB2023-014 deposited in Dalian Medical University Herbarium), *Scutellaria baicalensis* Georgi (Scutellariae Radix, origin: Liaoning Province, batch number 220527; validated via MPNS/POWO; voucher No. DMU-MB2023-015), *Senna alexandrina* Mill. (Sennae Folium, origin: Yunnan Province, batch number 220111; validated via MPNS/POWO; voucher No. DMU-MB2023-016), *Coptis chinensis* Franch. (Coptidis Rhizoma, origin: Sichuan Province, China, batch number 221027; validated via MPNS/POWO; voucher No. DMU-MB2023-017), *Codonopsis pilosula* (Franch.) Nannf. (Codonopsis Radix, origin: Jilin Province, batch number 220211; validated via MPNS/POWO; voucher No. DMU-MB2023-018), *Astragalus membranaceus* (Fisch.) Bunge (Astragali Radix, origin: Heilongjiang Province, batch number 220602; validated via MPNS/POWO; voucher No. DMU-MB2023-019), *Angelica sinensis* (Oliv.) Diels (Angelicae Sinensis Radix, origin: Sichuan Province, batch number 222203; validated via MPNS/POWO; voucher No. DMU-MB2023-020), *Panax ginseng* C.A.Mey. (Ginseng Radix et Rhizoma, origin: Liaoning Province, batch number 220301; validated via MPNS/POWO; voucher No. DMU-MB2023-021) were procured and authenticated by Prof. Li Tang (Dalian Medical University) in accordance with Chinese Pharmacopoeia (2020 Edition).

Ceftriaxone Sodium was acquired from Shandong Lukang Pharmaceutical Co., Ltd. (Jining, China). Test kits for Total Cholesterol (TC, batch 20230401), Triglyceride (TG, 20230311), High-Density Lipoprotein Cholesterol (HDL-C, 20230305), and Low-Density Lipoprotein Cholesterol (LDL-C, 20230315) were purchased from Nanjing Jiancheng Bioengineering Institute (Nanjing, China). The E. Z.N.A.^®^ Stool DNA kit was sourced from Omega (United States).

### 2.2 Analysis of effective metabolites

Chemical metabolites of the eight botanical drugs were retrieved from the Traditional Chinese Medicine Systems Pharmacology Database (TCMSP, https://old.tcmsp-e.com/tcmsp.php) following GA-online best practices (https://ga-online.org/best-practice/). Screening criteria included: Oral bioavailability (OB) ≥30% (threshold for intestinal absorption), Drug-likeness (DL) ≥0.18 (calculated via Tanimoto similarity), Half-life (HL) ≥4 h (estimated using the admetSAR 2.0 model). Identified metabolites were cross-validated against the Chinese Pharmacopoeia (2020 Edition) to confirm alignment with pharmacopeial standards (Liang et al., 2020).

### 2.3 Preparation of decoctions

The decoctions were prepared according to the standardized protocols outlined in the Chinese Pharmacopoeia (2020 Edition) to ensure consistency with clinical practice. Briefly, each authenticated botanical drug (Table 1) was soaked in distilled water at a drug-to-solvent ratio of 1:10 (w/v, g/mL) and maintained at 25°C ± 1°C for 1.5 h. The mixture was subsequently boiled at 100°C for 1 h under reflux conditions using a RE-52AA Rotary Evaporator (Yarong Biochemical Instrument Co., Shanghai, China). After filtration through sterile gauze (pore size: 100 μm; Millipore, USA), the residue was re-extracted with an additional 10 volumes of water under identical conditions. The combined filtrates were concentrated under reduced pressure (0.1 MPa, 60°C ± 2°C) using a Büchi R-300 rotary evaporator (Switzerland) to obtain lyophilized extracts (Zhao et al., 2021). The drug-extract ratios (DER, crude drug weight:lyophilized extract weight) were calculated and are listed in Table 1. Clinical-equivalent doses for mice were determined based on body surface area (BSA) conversion from human doses (Table 2), using the Reagan-Shaw formula: Animal dose = Human dose × (Human *K*_m_/Animal *K*_m_), where Human *K*_m_ = 37 and Mouse *K*_m_ = 3 (Reagan-Shaw et al., 2008). To ensure batch-to-batch consistency, key chemical markers (e.g., berberine in *Coptis chinensis* Franch. and ginsenoside Rh4 in *Panax ginseng* C.A. Mey.) were quantified via UPLC-Q-TOF-MS as described in Section 2.4.

**TABLE 1:** Drug-extract ratios (DER) of eight Chinese botanical drugs.

Botanical Drug (Latin Name)	DER (Crude Drug:Extract)
*Rheum palmatum* L.	6.7:1
*Scutellaria baicalensis* Georgi	10:1
*Senna alexandrina* Mill.	16.7:1
*Coptis chinensis* Franch.	20:1
*Codonopsis pilosula* (Franch.) Nannf.	3.3:1
*Astragalus membranaceus* (Fisch.) Bunge	3.3:1
*Angelica sinensis* (Oliv.) Diels	8.3:1
*Panax ginseng* C.A.Mey.	11.1:1

DER, was calculated as [crude drug weight]/[lyophilized extract weight].

**TABLE 2:** Animal dosage for individual botanical drugs (administered separately).

*Botanical Drug* (Latin Name)	Human dose (g/kg/day)	Animal dose (g/kg/day)
*Rheum palmatum* L.	0.06	0.75
*Scutellaria baicalensis* Georgi	0.04	0.5
*Senna alexandrina* Mill.	0.02	0.3
*Coptis chinensis* Franch.	0.02	0.3
*Codonopsis pilosula* (Franch.) Nannf.	0.12	1.5
*Astragalus membranaceus* (Fisch.) Bunge	0.12	1.5
*Angelica sinensis* (Oliv.) Diels	0.05	0.6
*Panax ginseng* C.A.Mey.	0.04	0.5

Human dose is based on the specified adult dosage in the 2020 edition of the Chinese Pharmacopoeia.

### 2.4 Ultra-high performance liquid tandemquadrupole time-of-flight mass spectrometry (UPLC-Q-TOF-MS) analysis

The chemical profiles of the botanical drug decoctions were analyzed using a Waters H-Class ultra-high-performance liquid chromatography (UPLC) system, along with an AB Sciex Triple TOF^®^ 4600 high-resolution mass spectrometer. Chromatographic separation was performed using a Waters CORTECS UPLC T3 column (2.1 × 100 mm, 1.6 µm) at a temperature of 30°C. The mobile phase consists of acetonitrile (A) and 0.1% formic acid aqueous solution (B), with a flow rate of 0.3 mL/min and an injection volume of 4 μL. Using gradient elution (Table 3).

**TABLE 3:** Gradient elution.

Time (min)	A%	B%
0∼3	3	97
3∼8	3∼10	97∼90
8∼30	10∼18	90∼82
30∼36	18∼30	82∼70
36∼41	30∼60	70∼40
41∼45	60∼95	40∼5
45∼47	95	5
47∼47.1	95∼3	5∼97
47.1∼50	3	97

The mass spectrometry detection mode was set to ESI-Negative/Positive ion mode, scanning a range of m/z 50 to 1,700. The ion source temperature was maintained at 500°C, with a declustering potential of 100 V and a collision energy of 10 eV. The curtain gas pressure was maintained at 35 psi, while the ion source Gas 1 (GS1) and the ion source Gas 2 (GS2) pressures were both set to 50 psi. UPLC-Q-TOF-MS data were collected.

### 2.5 Animal experiments

A total of 108 male, 8-week-old specific-pathogen free (SPF)-grade C57BL/6J mice weighing 20 ± 2 g were used as experimental animals. The mice were purchased from Liaoning Changsheng Biological Company (Benxi, China). Animal care and experimental procedures were approved by the Animal Ethics Committee of Dalian Medical University (No. AEE22210).

#### 2.5.1 Modeling and grouping of mice with gut microbiota dysbiosis

Fifty-four mice were randomly divided into nine groups, each consisting of six mice. The groups included the *Rheum palmatum* L. therapeutic gut microbiota dysbiosis group (A-DH), the *Scutellaria baicalensis* Georgi therapeutic gut microbiota dysbiosis group (A-HQ), the *Senna alexandrina* Mill. therapeutic gut microbiota dysbiosis group (A-FX), the *Coptis chinensis* Franch. therapeutic gut microbiota dysbiosis group (A-HL), the *Codonopsis pilosula* (Franch.) Nannf. therapeutic gut microbiota dysbiosis group (A-DS), the *Astragalus membranaceus* (Fisch.) Bunge therapeutic gut microbiota dysbiosis group (A-HU), the *Angelica sinensis* (Oliv.) Diels therapeutic gut microbiota dysbiosis group (A-DG), the *Panax Ginseng* C. A. Mey therapeutic gut microbiota dysbiosis group (A-RS), and the gut microbiota spontaneous recovery group (AN). To induce gut microbiota dysbiosis, all mice were administered 8 g/kg of ceftriaxone sodium daily for seven consecutive days (Zhao et al., 2020). After the 7-day of ceftriaxone sodium treatment, fecal samples were collected from all groups for DNA extraction and 16S rRNA sequencing analysis.

Following 7 days of ceftriaxone sodium administration via gavage, mice exhibiting dysbiosis were treated with botanical drug decoctions for 20 consecutive days. Prior to gavage, lyophilized extracts were reconstituted in distilled water to achieve target concentrations and sterilized using 0.22 μm microporous filters (Millipore, Billerica, MA, USA). All botanical drugs were administered at equal volumes (0.2 mL/mouse/day), with concentrations adjusted to achieve target doses (Table 2). No drug combinations were used across groups. The AN group received an equivalent volume of saline.

#### 2.5.2 Grouping of mice with normal gut microbiota

Another set of 54 mice was randomly divided into nine groups, with six mice in each group. These groups were designated based on the botanical drug they received: *Rheum palmatum* L. group (DH group), *Scutellaria baicalensis* Georgi group (HQ group), *Senna alexandrina* Mill. group (FX group), *Coptis chinensis* Franch. group (HL group), *Codonopsis pilosula* (Franch.) Nannf. group (DS group), *Astragalus membranaceus* (Fisch.) Bunge group (HU group), *Angelica sinensis* (Oliv.) Diels group (DG group), and *Panax Ginseng* C. A. Mey group (RS group). Additionally, there was a normal control group (NC group). Mice in each group were administered the corresponding decoctions for 20 consecutive days at the same concentrations as specified in Section 2.5.1. The NC group received an equivalent volume of physiological saline.

**FIGURE 1:**
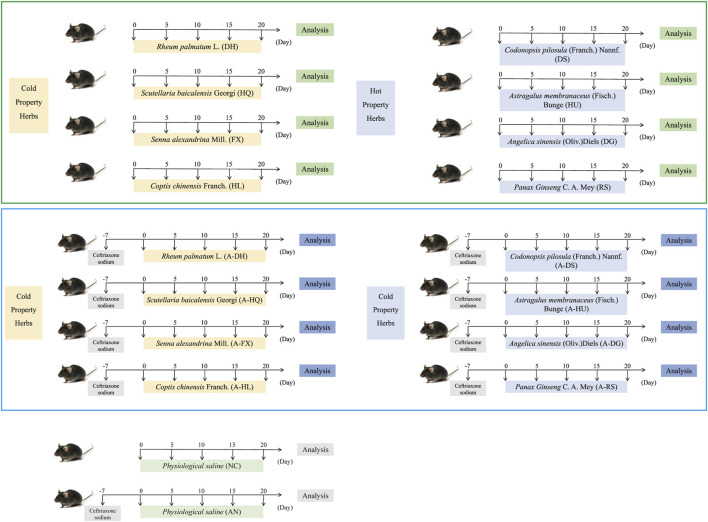
Specific grouping and experimental procedures for the experimental mice. Green boxes represent the normal model, while blue boxes represent the model of intestinal microflora disorder induced by ceftriaxone sodium.

The specific grouping and methods are shown in Figure 1.

### 2.6 Extraction and sequencing of total fecal microbiota DNA

After establishing dysbiosis models of gut microbiota, fecal samples (n = 6 for each group at each time point) were collected from mice in each group on the fifth, 10th, 15th, and 20th days following the oral administration of botanical drugs. Fecal DNA was extracted using an E. Z.N.A.^®^ Stool DNA kit (Omega, USA), and microbial 16S rRNA genes were targeted for amplification and purification of gene fragments in the V3-V4 region. The corresponding primers were 338F (5′-ACT​CCT​ACG​GGA​GGC​AGA​CG-3′) and 806R (5′-GGACTACHVGGGTWTCTAAT-3′). After sequencing was completed on the Illumina MiSeq PE300 platform (Majorbio, Shanghai, China), accurate high-quality and specific raw data were obtained by processing with QIIME (Version 1.9.1) noise reduction and optimization screening series. After comparing with the Silva database (https://www.arb-silva.de/), we annotated the taxonomic information of the species, calculated the species community composition of the samples, and performed operational unit clustering, colony diversity analysis, etc., on the measured valid data (Wang et al., 2020).

### 2.7 The determination of blood lipid content

After the experiment, the animals fasted for 14 h and were euthanized by intravenous injection of 90 mg/kg pentobarbital sodium solution. Collect whole blood samples (He et al., 2020). Then centrifuge the blood sample at 3,000 rpm for 15 min at 4°C and retain the supernatant. Following the manufacturer’s instructions, use kits to measure the concentrations of serum total cholesterol (TC), triglycerides (TG), high-density lipoprotein cholesterol (HDL-C), and low-density lipoprotein cholesterol (LDL-C) levels.

### 2.8 Determination of TNF-α and adiponectin content

According to the manufacturer’s instructions, use ELISA kits to measure the concentrations of TNF-α (Jiangsu Meibiao Biotechnology, YRMY-M000671A) and adiponectin (Jiangsu Meibiao Biotechnology, YRMY-M00712A) in the serum of each group.

### 2.9 Hematoxylin and eosin staining of intestinal tissues

Mouse jejunum and colon tissues were collected (n = 6 in each group), thoroughly rinsed in pre-cooled sterile PBS buffer, and then fixed in formalin for 24 h before being embedded in paraffin. Tissue sections, 5 μm thick, were stained with hematoxylin and eosin (Shen et al., 2023). Changes in the intestinal tissues were examined using an optical microscope, and histopathological scoring was employed to evaluate intestinal inflammation, lesion depth, crypt destruction, and lesion extent. The scoring criteria were as follows: inflammation (none = 0, mild = 1, moderate = 2, severe = 3); lesion depth (none = 0, mucosal layer = 1, submucosa = 2, muscle layer = 3, serosa layer = 4); crypt destruction (none = 0, base 1/3 = 1, base 2/3 = 2, only surface epithelium integrity = 3, total destruction = 4); lesion range (none = 0, 1%–25% = 1, 26%–50% = 2, 51%–75% = 3, 76%–100% = 4). The total severity score for the lesions was determined by summing the range and intensity scores for each specific lesion component (Qiu et al., 2023).

### 2.10 Statistical analysis

GraphPad Prism software version 9.0 (GraphPad, San Diego, CA, USA) is used for all statistical analyses. The results are reported as mean ± standard deviation (SD). To compare differences between two groups, unpaired Student t-tests are conducted. Mouse gut microbiota cluster analysis is performed to identify different flora patterns using ClusterViz in Cytoscape software (https://cytoscape.org/). A Spearman’s correlation analysis was conducted to compare the top 30 bacterial genera in the intestines of mice from each intervention group with those in the NC group at each intervention stage. This analysis served as a metric to assess the recovery of the gut microbiota in these mice. Additionally, at the 20-day mark of the experimental intervention, a Spearman’s correlation analysis was performed to examine the bacterial clusters of different model mice in relation to various botanical drugs. This analysis aimed to identify the characteristic bacterial clusters associated with botanical drugs of differing pharmacological properties. A *p*-value of <0.05 is deemed statistically significant.

## 3 Results

### 3.1 Determination of the properties of eight botanical drugs

According to the traditional classification system outlined in the Chinese Pharmacopoeia (2020 Edition), we selected four botanical drugs categorized as “cold” (*Rheum palmatum* L., *Scutellaria baicalensis* Georgi, *Senna alexandrina* Mill., *Coptis chinensis* Franch.) and four as “hot” (*Codonopsis pilosula* (Franch.) Nannf., *Astragalus membranaceus* (Fisch.) Bunge, *Angelica sinensis* (Oliv.) Diels, *Panax ginseng* C.A. Mey.) based on their documented therapeutic effects in TCM theory (Xu et al., 2019). Their traditional properties are summarized in Table 4.

**TABLE 4:** TCM-classified cold and hot properties of single botanical drugs.

Botanical Drug Names	Latin Name	Family name	Nature
Da huang	*Rheum palmatum* L.	Polygonaceae	Cold
Huang qin	*Scutellaria baicalensis* Georgi	Labiatae	Cold
Fan xie ye	*Senna alexandrina* Mill.	Fabaceae	Cold
Huang lian	*Coptis chinensis* Franch.	Ranunculaceae	Cold
Dang shen	*Codonopsis pilosula* (Franch.) Nannf.	Campanulaceae	Hot
Huang qi	*Astragalus membranaceus* (Fisch.) Bunge	Fabaceae	Hot
Dang gui	*Angelica sinensis* (Oliv.)Diels	Umbelliferae	Hot
Ren shen	*Panax ginseng* C. A. Mey.	Araliaceae	Hot

### 3.2 Identification of chemical profiles and predicted bioactive compounds in eight botanical drugs

First, UPLC-Q-TOF-MS was employed to determine the chemical profiles of the botanical drug decoctions. The specific mass spectra and quantitative values of major compounds are provided in Supplementary Material S1 (Supplementary Figure S1; Supplementary Table S1). Subsequently, potential bioactive compounds were screened via the Traditional Chinese Medicine Systems Pharmacology Database (TCMSP) using the following criteria: oral bioavailability (OB) ≥30%, drug-likeness (DL) ≥0.18, and half-life (HL) ≥4 h. From *Rheum palmatum* L., *Scutellaria baicalensis* Georgi, *Senna alexandrina* Mill., *Coptis chinensis* Franch., *Codonopsis pilosula* (Franch.) Nannf., *Astragalus membranaceus* (Fisch.) Bunge, *Angelica sinensis* (Oliv.)Diels, and *Panax Ginseng* C. A. Mey, we identified 17, 39, 11, 12, 21, 19, 2, and 24 active chemical metabolites, respectively, as detailed in Supplementary Material S1 (Supplementary Table S2). Notably, cold-classified drugs (*Coptis chinensis* Franch., *Rheum palmatum* L., etc.) exhibited higher proportions of alkaloids (e.g., *berberine*, OB = 36.86%) and monoterpenes, while hot-classified drugs (*Panax ginseng* C.A. Mey., *Astragalus membranaceus* (Fisch.) Bunge, etc.) were enriched with saponins (e.g., ginsenoside-Rh4, OB = 31.11%) and phenylpropanoids (Supplementary Tables S2, S3). These phytochemical trends align with prior reports linking TCM thermal properties to compound classes (Peng et al., 2023).

### 3.3 Establishment of a mouse model for gut microbiota dysbiosis

**FIGURE 2:**
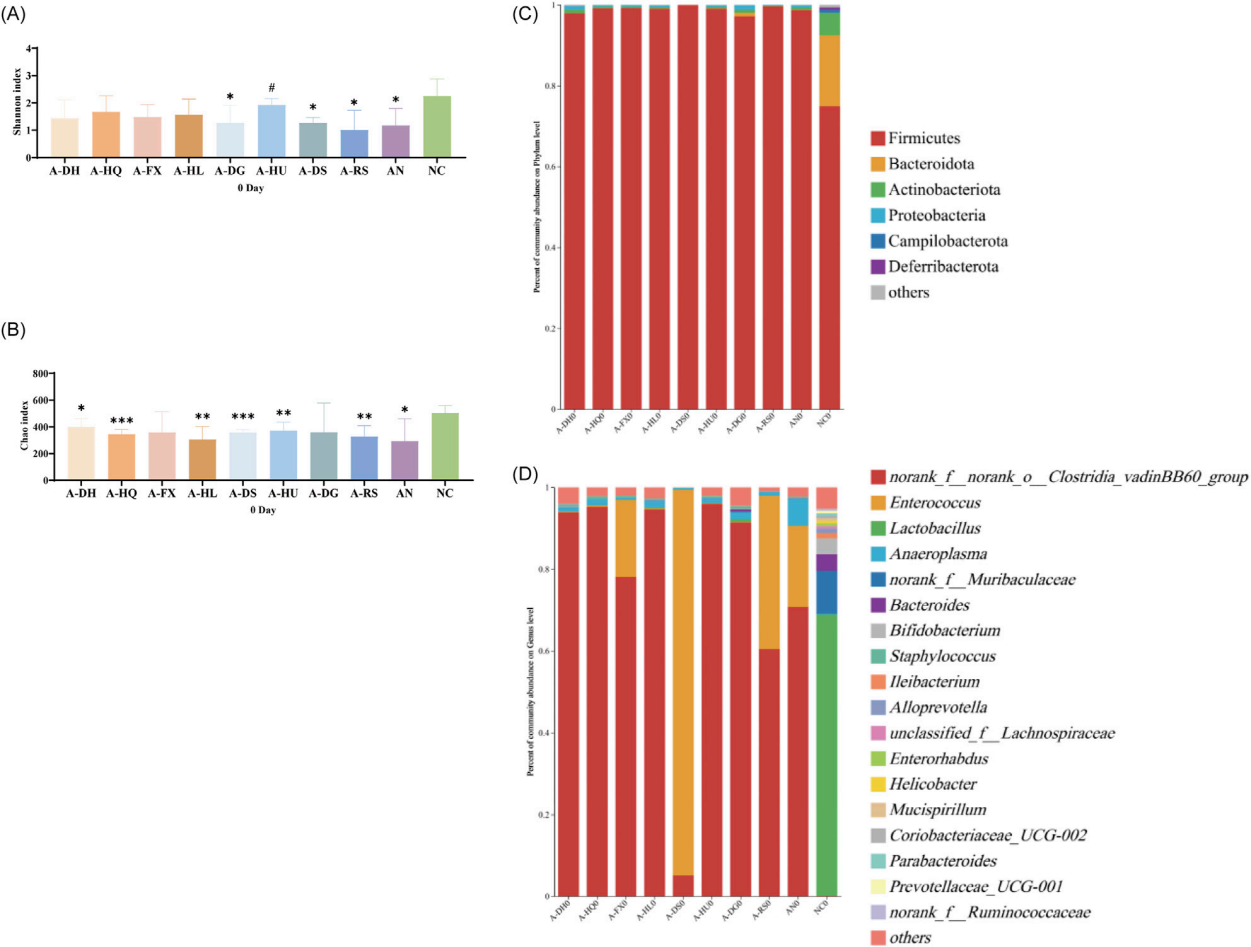
Establishment of a mouse model with disrupted gut microbiota induced by ceftriaxone sodium: **(A)** Shannon Index, **(B)** Chao Index, **(C)** Analysis of phylum-level community composition, **(D)** Analysis of genus-level community composition. Compared to the NC group, * indicates *P* < 0.05, ** indicates *P* < 0.01, and *** indicates *P* < 0.001. Additionally, # indicates *P* < 0.05 when compared to the AN group.

In order to elicit gut microbiota dysbiosis, ceftriaxone sodium was orally administered to the mice. After 1 week of continuous administration, significant changes in the gut microbiota were observed compared to the normal control (NC) group. Alpha diversity analysis revealed a decrease in species diversity and richness (*P* < 0.05). The Shannon index exhibited significant decreases in the A-DG (*P* < 0.05), A-DS(*P* < 0.05), A-RS(*P* < 0.05), and AN groups (*P* < 0.05). Meanwhile, the Chao index showed significant reductions in the A-DH(*P* < 0.05), A-HQ (*P* < 0.001), A-HL (*P* < 0.01), A-DS(*P* < 0.001), A-HU(*P* < 0.01), A-RS(*P* < 0.01), and AN groups (*P* < 0.05) (Figures 2A,B). Principal Coordinate Analysis (PCoA) plots demonstrated a distinct separation in the composition of gut microbiota between the NC group and the antibiotic-treated groups (Supplementary Figure S2). The gut microbiota of mice in all antibiotic-treated groups was dominated by Firmicutes, with *Clostridia vadinBB60 group* or *Enterococcus* being the absolute predominant genera during this period (Figures 2C,D). Compared to the NC group, the antibiotic-intervened mice exhibited decreased alpha diversity, significantly different community compositions, and a simplified structure of dominant genera within the gut microbiota, indicating the successful establishment of the model.

### 3.4 Impact of botanical drugs on gut microbiota dysbiosis induced by ceftriaxone sodium in mice at various intervention time points

#### 3.4.1 Analysis of gut microbiota in mice following 5 days of botanical drug intervention

**FIGURE 3:**
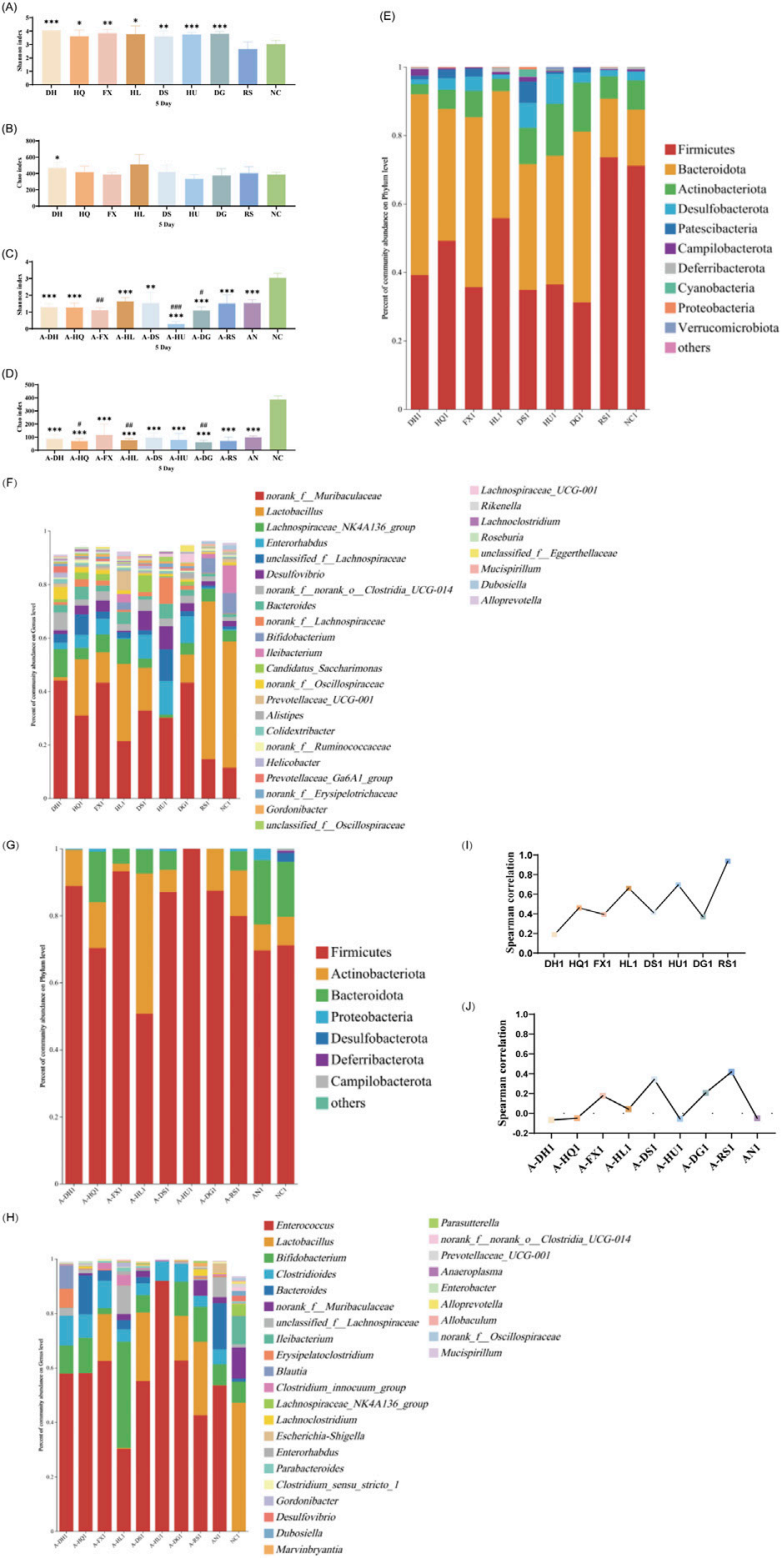
Effect of a 5-Day Botanical Drug Intervention on the Gut Microbiota of Mice: **(A)** Shannon Index under Normal Conditions. **(B)** Chao Index under Normal Conditions. **(C)** Shannon Index in the Context of Gut Microbiota Dysbiosis. **(D)** Chao Index in the Context of Gut Microbiota Dysbiosis. **(E)** Analysis of Phylum-Level Community Composition under Normal Conditions. **(F)** Analysis of Genus-Level Community Composition under Normal Conditions. **(G)** Analysis of Phylum-Level Community Composition in the Context of Gut Microbiota Dysbiosis. **(H)** Analysis of Genus-Level Community Composition in the Context of Gut Microbiota Dysbiosis. **(I)** Genus-Level Correlation Analysis under Normal Conditions. **(J)** Genus-Level Correlation Analysis in the Context of Gut Microbiota Dysbiosis. Compared with the NC group, * indicates *P* < 0.05, ** indicates *P* < 0.01, and *** indicates *P* < 0.001. Compared with the AN group, # indicates *P* < 0.05, ## indicates *P* < 0.01, and ### indicates *P* < 0.001.

An α-diversity analysis of the 16S rRNA sequences of the gut microbiota in mice was conducted. Under normal conditions, both cold and hot botanical drugs significantly increased the Shannon index of the gut microbiota in mice (*P* < 0.05) (except for the RS group), while the Chao index did not significantly differ from that of the NC group (except for the DH group, *P* < 0.05) (Figures 3A,B). PCoA plot analysis revealed a low degree of dispersion between the gut microbiota of the RS group and the NC group, while other groups showed significant differences (*P* < 0.05) (Supplementary Figure S3). To delve into the key impacts of different TCM-classified of botanical drugs on the gut microbiota at this stage, we performed a detailed analysis of the microbiota composition at the phylum and genus levels in each group and conducted a correlation analysis between the top 30 most abundant genera in each intervention group and the NC group. Species composition analysis indicated that the RS group had a relatively minor impact on the gut microbiota, whereas both cold and hot-classified drugs (except the RS group) tended to decrease the abundance of Firmicutes and promote the growth of Bacteroidetes. Additionally, hot-classified drugs also exhibited a promoting effect on Actinobacteriota (Figure 3E). At the genus level, all groups except the RS group showed inhibition of *Lactobacillus* and promoted the growth of *Muribaculaceae* (Figure 3F). Correlation analysis further confirmed that both types of botanical drugs (except the RS group) had significant impacts on the gut microbiota (*R* < 0.8), with cold-classified drugs exerting relatively stronger effects (Figure 3I).

In the context of gut microbiota dysbiosis, the natural recovery group (AN group) receiving antibiotic intervention showed significantly decreased Shannon and Chao indices compared to the NC group (*P* < 0.05), indicating a pronounced negative effect of antibiotics on the gut microbiota. After the intervention with the two types of TCM-classified botanical drugs, the Shannon index further decreased in the A-FX (*P* < 0.01), A-HU (*P* < 0.001), and A-DG groups (*P* < 0.05). The Chao index of the A-HQ (*P* < 0.05), A-HL (*P* < 0.01), and A-DG (*P* < 0.01) groups was significantly different from that of the AN group, with a more pronounced decrease observed in the hot-classified drug groups compared to the cold-classified drug groups (Figures 3C,D). PCoA plot analysis indicated that the gut microbiota of the two types of botanical drugs groups began to separate from that of the AN group, with a stronger degree of dispersion observed in the hot-classified drug groups (Supplementary Figure S4). At this stage, the gut microbiota of the AN group was dominated by Firmicutes and Bacteroidetes, with *Enterococcus*, *Bacteroides*, *Bifidobacterium*, and *Clostridioides* being the dominant genera. All botanical drugs intervention groups inhibited the growth of *Bacteroidetes*. Except for the A-HL group, other botanical drugs intervention groups showed a growth advantage of *Enterococcus*. Furthermore, cold-classified drugs primarily promoted the growth of *Bifidobacterium* (except the A-FX group), while hot botanical drugs mainly promoted the growth of *Lactobacillus* and *Bifidobacterium* (except the A-HU group) (Figures 3G,H). Correlation analysis at the genus level revealed that the gut microbiota of mice in the AN group was severely disrupted (*R* < 0.8), and 5 days of TCM intervention had limited restorative effects (Figure 3J).

#### 3.4.2 Analysis of gut microbiota in mice following a 10-day botanical drug intervention

**FIGURE 4:**
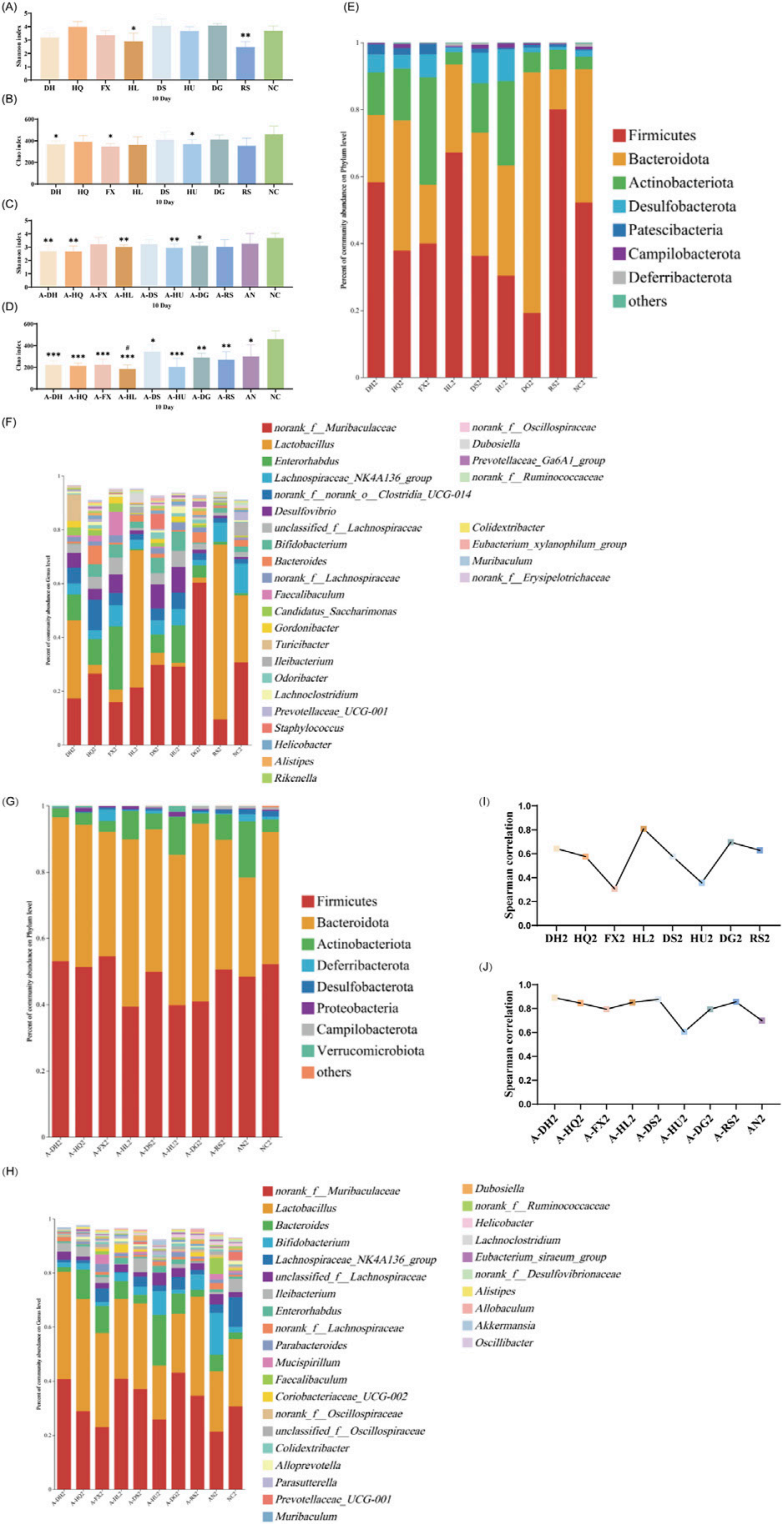
Effect of a 10-Day Botanical Drug Intervention on the Gut Microbiota of Mice: **(A)** Shannon Index under Normal Conditions. **(B)** Chao Index under Normal Conditions. **(C)** Shannon Index in the Context of Gut Microbiota Dysbiosis. **(D)** Chao Index in the Context of Gut Microbiota Dysbiosis. **(E)** Analysis of Phylum-Level Community Composition under Normal Conditions. **(F)** Analysis of Genus-Level Community Composition under Normal Conditions. **(G)** Analysis of Phylum-Level Community Composition in the Context of Gut Microbiota Dysbiosis. **(H)** Analysis of Genus-Level Community Composition in the Context of Gut Microbiota Dysbiosis. **(I)** Genus-Level Correlation Analysis under Normal Conditions. **(J)** Genus-Level Correlation Analysis in the Context of Gut Microbiota Dysbiosis. Compared with the NC group, * indicates *P* < 0.05, ** indicates *P* < 0.01, and *** indicates *P* < 0.001. Compared with the AN group, # indicates *P* < 0.05.

As depicted in Figure 4, after 10 days of botanical drugs intervention in normal mice, the α-diversity of fecal microbiota in the HQ and DS groups did not significantly differ from that of the NC group. The Shannon index in the HL group (*P* < 0.05) and the RS group (*P* < 0.01) showed significant decreases, while the Chao index in the DH group (*P* < 0.05), the FX group (*P* < 0.05), and the HU group (*P* < 0.05) exhibited notable reductions. No significant differences were observed in the effects of different TCM-classified botanical drugs on the α-diversity of gut microbiota in mice (Figures 4A,B). PCoA plot revealed that the dispersion between the gut microbiota of mice in the hot-classified drug groups and that of normal mice was more pronounced compared to the cold-classified drug groups (Supplementary Figure S5). From the species composition and genus correlation analysis, we also failed to observe significant differences in the impacts of the two types of botanical drugs on the gut microbiota of normal mice. The abundance of *Lactobacillus* in the intestines of mice in the HL and RS groups increased compared to the NC group, and the gut microbiota composition of the HL group was closest to that of the NC group (Figures 4E,F,I).

Under the condition of gut microbiota dysbiosis, after 10 days of botanical drugs intervention, the α-diversity of fecal microbiota in all intervention groups and the AN group still significantly differed from that of the NC group. Compared to the AN group, the community richness (Chao index) in the A-HL group significantly decreased (*P* < 0.05), while no significant differences were observed in other intervention groups (Figures 4C,D). PCoA showed that the recovery of gut microbiota in all intervention groups was significantly better than that in the AN group, with a distribution closer to the NC group (Supplementary Figure S6). Compared to the NC group, Bacteroidetes decreased while Actinobacteriota increased in the intestines of mice in the AN group at this stage. Both cold and hot-classified botanical drugs exhibited a trend of promoting Bacteroidetes growth and inhibiting Actinobacteriota growth (Figure 4G). Among all intervention groups, *Lactobacillus* and *Muribaculaceae* dominated in abundance, with the lowest abundance sum observed in the AN group, intermediate in the NC group, and higher levels in all botanical drugs intervention groups (except the A-HU group) (Figure 4H). Correlation studies further indicated that after 10 days of intervention, the disrupted gut microbiota of mice had undergone preliminary restoration, with cold-classified drugs showing a more pronounced effect (*R* > 0.8) (Figure 4J).

#### 3.4.3 Analysis of gut microbiota in mice following a 15-day botanical drug intervention

**FIGURE 5:**
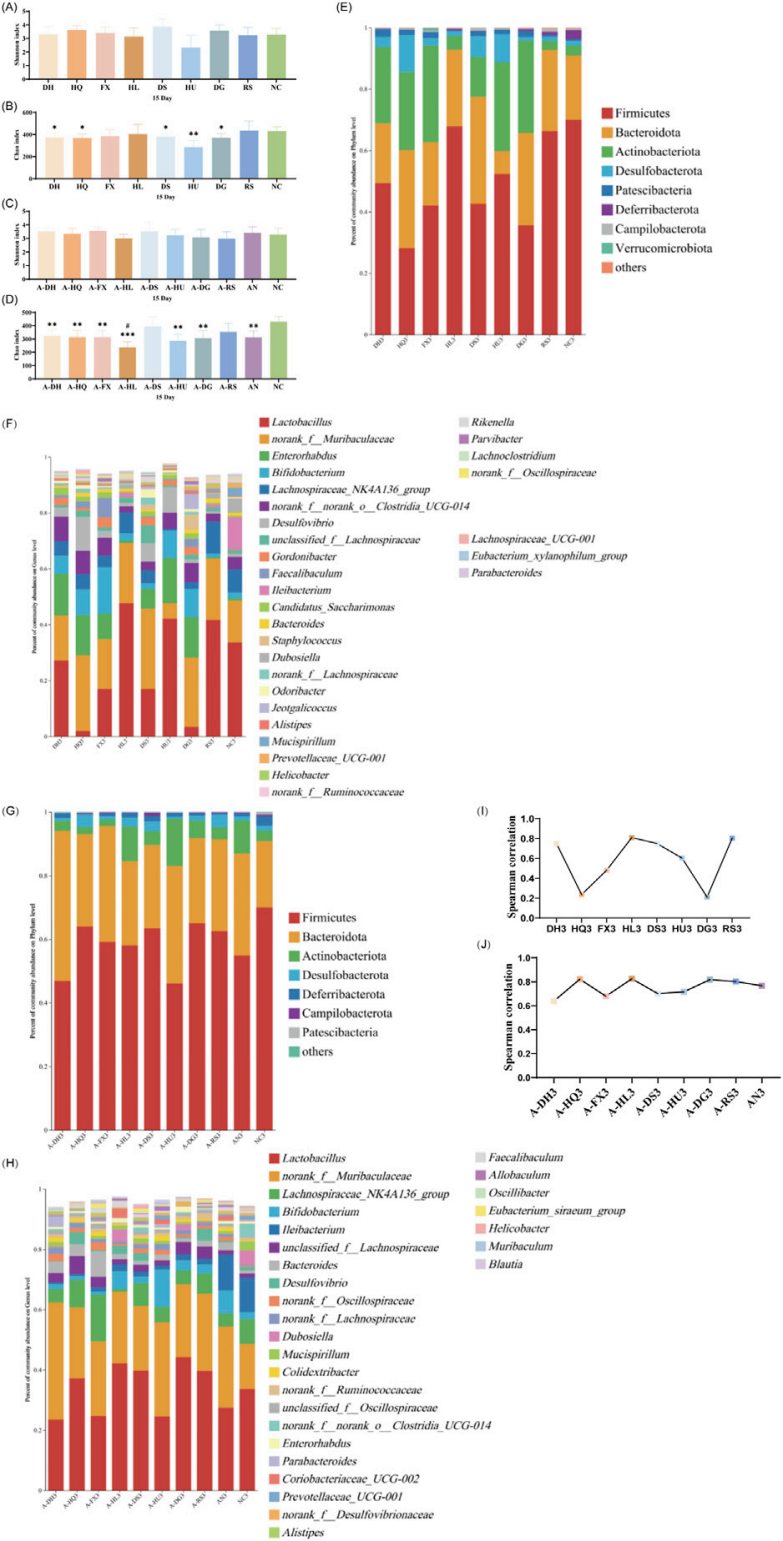
Effect of a 15-Day Botanical Drug Intervention on the Gut Microbiota of Mice: **(A)** Shannon Index under Normal Conditions. **(B)** Chao Index under Normal Conditions. **(C)** Shannon Index in the Context of Gut Microbiota Dysbiosis. **(D)** Chao Index in the Context of Gut Microbiota Dysbiosis. **(E)** Analysis of Phylum-Level Community Composition under Normal Conditions. **(F)** Analysis of Genus-Level Community Composition under Normal Conditions. **(G)** Analysis of Phylum-Level Community Composition in the Context of Gut Microbiota Dysbiosis. **(H)** Analysis of Genus-Level Community Composition in the Context of Gut Microbiota Dysbiosis. **(I)** Genus-Level Correlation Analysis under Normal Conditions. **(J)** Genus-Level Correlation Analysis in the Context of Gut Microbiota Dysbiosis. Compared with the NC group, * indicates *P* < 0.05, ** indicates *P* < 0.01, and *** indicates *P* < 0.001. Compared with the AN group, # indicates *P* < 0.05.

As depicted in Figure 5, on the 15th day post-botanical drugs intervention, no significant differences in the Shannon index were observed among the gut microbiota of mice in various groups compared to the NC group (Figure 5A). However, significant differences in the Chao index were noted between the DH (*P* < 0.05), HQ (*P* < 0.05), DS (*P* < 0.05), HU (*P* < 0.05), and DG (*P* < 0.05) groups and the NC group (Figure 5B). Principal component analysis revealed that the microbial structures in the HL and RS groups did not deviate significantly from that of the NC group, while the gut microbiota structures of other intervention groups and the NC group mice exhibited clear separation on the PCoA plot (Supplementary Figure S7). Species composition analysis showed further restoration of gut microbiota in the HL and RS groups, with their microbiota compositions becoming more similar to that of the NC group. Cold-classified drugs (excluding the HL group) significantly promoted the growth of bacteria such as *Clostridia UCG 014*, *Enterorhabdus*, and *Bifidobacterium*, while also narrowing the abundance gap of *Muribaculaceae* compared to the NC group (Figures 5E,F). Correlation analysis revealed significant disparities in the regulatory effects of the eight botanical drugs on gut microbiota in mice. The R-coefficients of these botanical drugs demonstrated substantial variability, with the four botanical drugs of the same thermal nature (either cold or hot-classified) exhibiting fluctuations between 0.2 and 0.8. (Figure 5I).

After 15 days of natural recovery, mice in the AN group with gut microbiota dysbiosis exhibited a restored Shannon index to normal levels, while the Chao index remained significantly lower than that of the NC group. The Chao indices of all botanical drug intervention groups were notably lower than those of the NC group (*P* < 0.05), with the exception of the A-DS and A-RS groups. Furthermore, the Chao index in the A-HL group was significantly lower than that in the AN group (*P* < 0.05) (Figures 5C,D). PCoA indicated reduced dispersion among the gut microbiota structures of the intervention groups, the AN group, and the NC group, suggesting improved stability of the gut microbiota (Supplementary Figure S8). At this stage, the gut microbiota of mice in the AN group and all intervention groups were effectively restored at the phylum level (Figure 5G). At the genus level, *Lactobacillus*, *Muribaculaceae*, and *Ileibacterium* showed the highest abundances in the intestines of mice in the AN and NC groups; however, in the intervention groups, *Ileibacterium* growth was suppressed, with *Lactobacillus* and *Muribaculaceae* dominating the gut microbiota (Figure 5H). Correlation analysis indicated that after 15 days of botanical drugs intervention, the microbiota structure of mice with intestinal dysbiosis was effectively restored (*R* > 0.6), with no disparity in recovery efficacy between the two types of TCM-classified botanical drugs. Notably, *Scutellaria baicalensis* Georgi (*R >* 0.8) and *Coptis chinensis* Franch. (*R* > 0.8) demonstrated the most effective recovery effects (Figure 5J).

#### 3.4.4 Analysis of gut microbiota in mice following a 20-day botanical drug intervention

**FIGURE 6:**
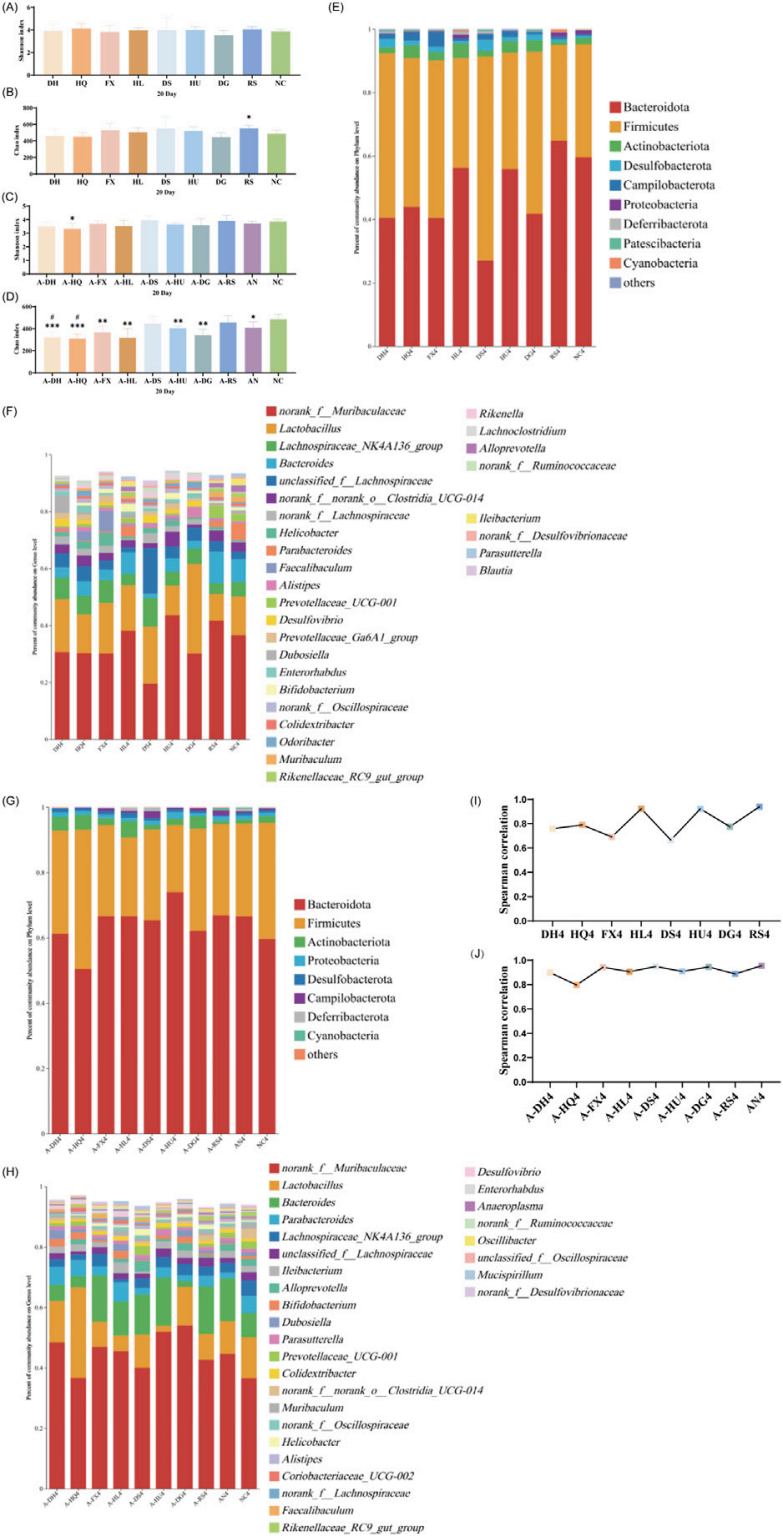
Effect of a 20-Day Botanical Drug Intervention on the Gut Microbiota of Mice: **(A)** Shannon Index under Normal Conditions. **(B)** Chao Index under Normal Conditions. **(C)** Shannon Index in the Context of Gut Microbiota Dysbiosis. **(D)** Chao Index in the Context of Gut Microbiota Dysbiosis. **(E)** Analysis of Phylum-Level Community Composition under Normal Conditions. **(F)** Analysis of Genus-Level Community Composition under Normal Conditions. **(G)** Analysis of Phylum-Level Community Composition in the Context of Gut Microbiota Dysbiosis. **(H)** Analysis of Genus-Level Community Composition in the Context of Gut Microbiota Dysbiosis. **(I)** Genus-Level Correlation Analysis under Normal Conditions. **(J)** Genus-Level Correlation Analysis in the Context of Gut Microbiota Dysbiosis. Compared with the NC group, * indicates *P* < 0.05, ** indicates *P* < 0.01, and *** indicates *P* < 0.001. Compared with the AN group, # indicates *P* < 0.05.

After 20 days of intervention, only the Chao index of the gut microbiota in the RS group mice significantly increased (*P* < 0.05), while the α-diversity of the other groups did not differ statistically from the NC group (Figures 6A,B). PCoA revealed a further reduction in the microbial dispersion between the botanical drugs intervention groups and the NC group, indicating that the gut microbiota structures of mice in these groups gradually stabilized under intervention (Supplementary Figure S9). Species composition analysis found that among the gut microbiota of mice in each group, there were minor differences in bacteria with higher species richness, while the primary differences were observed in bacteria with lower abundances. The gut microbiota changes in mice from the cold-classified drug groups displayed consistency across different herbal flavors, while those in the hot-classified drug groups still exhibited a certain degree of variability (Figures 6E,F). Correlation analysis at the genus level showed that all eight botanical drugs had a stabilizing effect on the gut microbiota of normal mice (*R* > 0.6), and their regulatory effects on gut microbiota did not exhibit distinct trends based on their TCM-classified (Figure 6I).

In the intestinal dysbiosis model, the Chao index of the gut microbiota in mice after 20 days of natural recovery was significantly lower than that of the NC group (*P* < 0.05). However, after botanical drugs intervention, only the A-DS and A-RS groups showed no significant difference in α-diversity compared to the NC group. The Chao index in the A-DH (*P* < 0.001), A-HQ (*P* < 0.001), A-FX (*P* < 0.01), A-HL (*P* < 0.01), A-HU (*P* < 0.01), and A-DG (*P* < 0.001) groups remained significantly lower than that in the NC group (Figures 6C,D). PCoA revealed that the experimental groups with botanical drugs intervention were closer to the AN and NC groups in microbial distribution, indicating a reduced difference in microbiota structure among the three groups, which suggests the positive impact of botanical drugs intervention on the restoration of gut microbiota structure (Supplementary Figure S10). Species composition and correlation analysis found that the gut microbiota of mice in all groups were effectively restored at this stage (*R* > 0.8). Nevertheless, the recovery effects of the gut microbiota in the intervention groups did not significantly surpass that of the AN group (Figures 6G,H,J).

Supplementary Figures S11, S12 illustrate the genus-level composition of gut microbiota in mice after 20 days of intervention with single botanical drugs under both normal and dysbiotic conditions, respectively. Quantitative analysis of dominant genera (defined as taxa with >10% relative abundance) revealed that both cold and hot-classified drugs stabilized *Lactobacillus* (normal model: 16.56% in cold botanical drugs vs. 17.85% in hot botanical drugs; dysbiosis model: 13.88% vs. 8.02%) and *Muribaculaceae* (normal: 32.27% vs. 33.67%; dysbiosis: 44.58% vs. 47.02%), with no statistical differences between TCM-classified (Tables 5 and 6).

**TABLE 5:** Composition ratio of mouse gut microbiota after different types of Botanical Drug interventions (Normal model).

Composition ratio	Cold Botanical Drug (A group)	Hot Botanical Drug (B group)	NC group
30%–40%	*norank_f__Muribaculaceae*	*norank_f__Muribaculaceae*	*norank_f__Muribaculaceae*
20%–30%	——	——	——
10%–20%	*Lactobacillus*	*Lactobacillus*	*Lactobacillus*
1%–10%	*Lachnospiraceae_NK4A136_group* *Bacteroides unclassified_f__Lachnospiraceae* *norank_f__norank_o__Clostridia_UCG-014* *Faecalibaculum* *Helicobacter* *Dubosiella norank_f__Lachnospiraceae* *Prevotellaceae_Ga6A1_group* *Enterorhabdus* *Desulfovibrio* *Parabacteroides* *Bifidobacterium* *Alistipes norank_f__Oscillospiraceae*	*unclassified_f__Lachnospiraceae* *Lachnospiraceae_NK4A136_group* *Bacteroides norank_f__norank_o__Clostridia_UCG-014* *norank_f__Lachnospiraceae* *Alistipes* *Desulfovibrio* *Helicobacter* *Prevotellaceae_UCG-001* *Odoribacter* *Prevotellaceae_Ga6A1_group* *Enterorhabdus*	*Bacteroides* *Parabacteroides* *Lachnospiraceae_NK4A136_group norank_f__norank_o__Clostridia_UCG-014* *unclassified_f__Lachnospiraceae* *Prevotellaceae_UCG-001* *Alloprevotella* *Ileibacterium* *Muribaculum* *Rikenellaceae_RC9_gut_group* *Parasutterella* *Dubosiella* *Bifidobacterium*
≤1%	*Colidextribacter* *Prevotellaceae_UCG-001* *Odoribacter* *Muribaculum* *Lachnoclostridium norank_f__Ruminococcaceae* *Rikenellaceae_RC9_gut_group* others	*Rikenella* *Bifidobacterium norank_f__Oscillospiraceae* *Colidextribacter* *Muribaculum* *Lachnoclostridium* *Parabacteroides* *Blautia* *Rikenellaceae_RC9_gut_group norank_f__Ruminococcaceae* others	*Colidextribacter* *Alistipes norank_f__Desulfovibrionaceae* others

**TABLE 6:** Composition ratio of mouse gut microbiota after different types of Botanical Drug interventions (Model of gut microbiota disorder caused by ceftriaxone sodium).

Composition ratio	Cold Botanical Drug (AA group)	Hot Botanical Drug (AB group)	AN group	NC group
40%–50%	*norank_f__Muribaculaceae*	*norank_f__Muribaculaceae*	*norank_f__Muribaculaceae*	—
30%–40%	—	—	—	*norank_f__Muribaculaceae*
20%–30%	—	—	—	—
10%–20%	*Lactobacillus*	*Bacteroides*	*Bacteroides* *Lactobacillus*	*Lactobacillus*
1%–10%	*Bacteroides* *Parabacteroides* *Ileibacterium* *Lachnospiraceae_NK4A136_group* *Bifidobacterium unclassified_f__Lachnospiraceae* *Dubosiella* *Parasutterella*	*Lactobacillus* *Lachnospiraceae_NK4A136_group* *Parabacteroides unclassified_f__Lachnospiraceae* *Alloprevotella* *Ileibacterium* *Helicobacter* *Parasutterella* *Prevotellaceae_UCG-001* *Colidextribacter*	*Lachnospiraceae_NK4A136_group* *Ileibacterium* *Alloprevotella* *Parabacteroides* *Prevotellaceae_UCG-001 unclassified_f__Lachnospiraceae* *Parasutterella* *Colidextribacter*	*Bacteroides* *Parabacteroides* *Lachnospiraceae_NK4A136_group norank_f__norank_o__Clostridia_UCG-014* *unclassified_f__Lachnospiraceae* *Prevotellaceae_UCG-001* *Alloprevotella* *Ileibacterium* *Muribaculum* *Rikenellaceae_RC9_gut_group* *Parasutterella* *Dubosiella* *Bifidobacterium*
≤1%	*Colidextribacter* *Coriobacteriaceae_UCG-002 norank_f__Oscillospiraceae* *Desulfovibrio* *Alloprevotella* *Faecalibaculum* *Muribaculum norank_f__norank_o__Clostridia_UCG-014* *Enterorhabdus* *Prevotellaceae_UCG-001* *Alistipes norank_f__Lachnospiraceae* others	*Bifidobacterium* *Dubosiella norank_f__norank_o__Clostridia_UCG-014* *Muribaculum norank_f__Oscillospiraceae* *Alistipes norank_f__Lachnospiraceae* *Rikenellaceae_RC9_gut_group* *Faecalibaculum* others	*Alistipes norank_f__Oscillospiraceae* *Mucispirillum norank_f__norank_o__Clostridia_UCG-014* *norank_f__Lachnospiraceae* *Dubosiella norank_f__Desulfovibrionaceae* *Rikenellaceae_RC9_gut_group norank_f__Ruminococcaceae* others	*Colidextribacter* *Alistipes norank_f__Desulfovibrionaceae* others

### 3.5 Differential enrichment of gut microbiota taxa in response to botanical drugs classified as cold or hot in TCM

**FIGURE 7:**
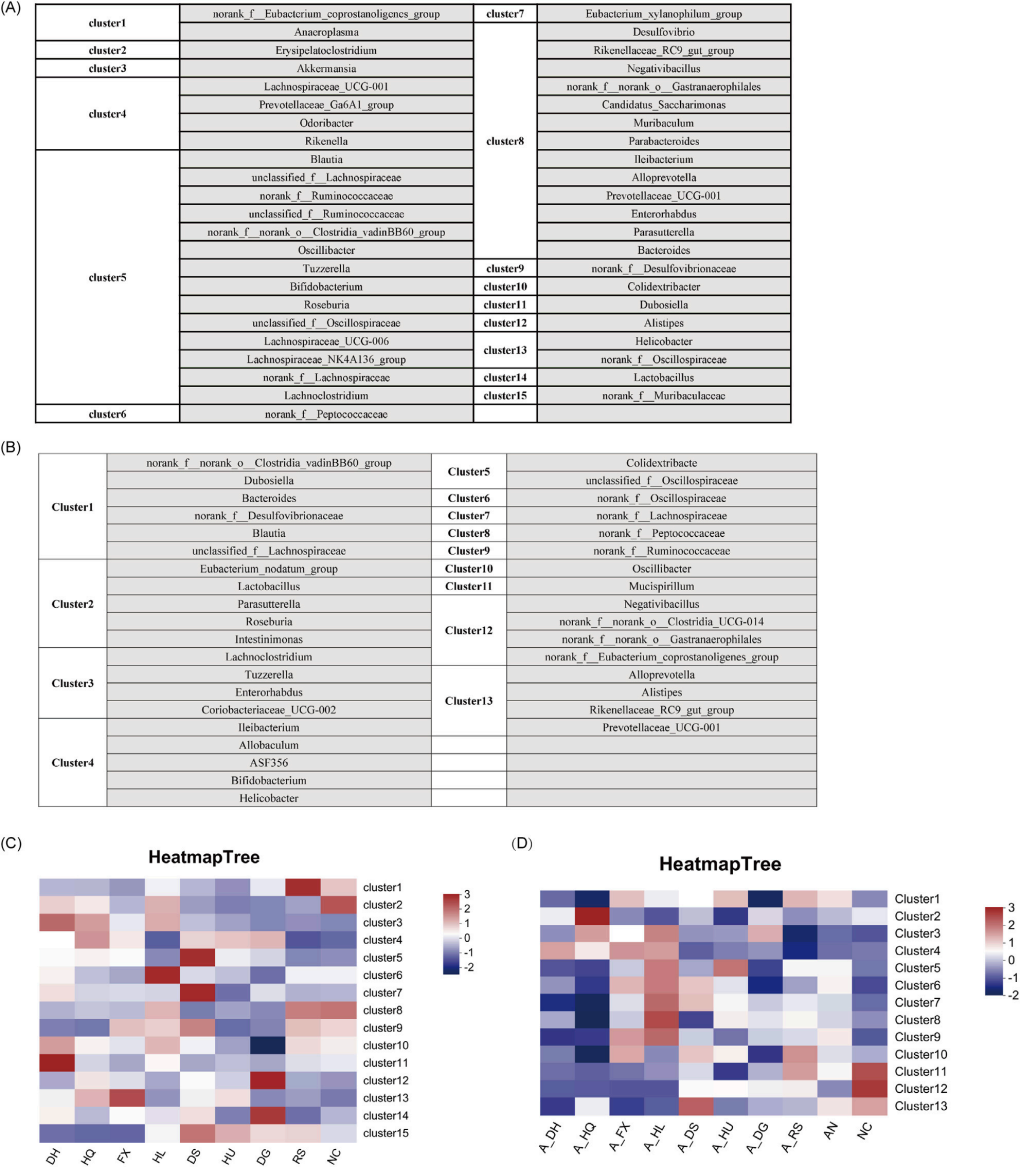
Characteristics of Gut Microbiota Associated with Various Botanical Drugs: **(A)** Identification of Gut Microbiota Clusters Based on ClusterViz Analysis Under Normal Conditions. **(B)** Identification of Gut Microbiota Clusters Based on ClusterViz Analysis in the Context of Gut Microbiota Dysbiosis. **(C)** Correlation Analysis of Each Cluster with Different Botanical Drugs Under Normal Conditions. **(D)** Correlation Analysis of Each Cluster with Different Botanical Drugs in the Context of Gut Microbiota Dysbiosis. Note: Microbial clusters were analyzed based on the eight selected botanical drugs. Generalizability to other TCM-classified drugs requires further investigation.

Utilizing the ClusterViz analysis tool integrated within Cytoscape software, we investigated the distinct effects of cold and hot-classified drugs on gut microbiota across various microbiota profiles. Initially, correlation analyses were conducted on the 50 most prevalent bacterial genera found in the intestines of mice, including both normal and ceftriaxone sodium-induced dysbiosis models. Genera with an absolute correlation coefficient (|R|) of at least 0.5 and a *P*-value of less than 0.05 were selected for subsequent clustering analysis. The findings revealed that the gut microbiota of normal model mice could be categorized into 15 primary clusters (Figure 7A), while those of mice with microbiota dysbiosis formed 13 main clusters (Figure 7B). By linking these clusters with specific botanical drugs, we observed that under normal conditions, the gut microbiota in Cluster 3 (e.g., *Akkermansia*) exhibited a significant positive correlation with cold-classified drugs (91.6 ± 15.2 vs. 0 in NC group, *P* < 0.01), whereas the microbiota in Cluster 15 (e.g., *Muribaculaceae*) showed a significant positive correlation with hot-classified drugs (17981.4 ± 545.8 vs. 10283.15 ± 1851.5 in NC group, *P* < 0.01) (Figure 7C). In the dysbiosis state, the gut microbiota in Cluster 4 (*Allobaculum*, *Bifidobacterium*, etc.) displayed a significant positive correlation with cold-classified drugs (1,633.25 ± 162.5 vs. 615.3 ± 199.1 in AN group, *P* < 0.05), while the microbiota in Cluster 12 (e.g., *Clostridia*, *Eubacterium coprostanoligenes*) demonstrated a significant positive correlation with hot-classified drugs (379.95 ± 21.42 vs. 113.4 ± 86.4 in AN group, *P* < 0.05) (Figure 7D). This differential microbial response aligns with TCM’s empirical classification, though further validation is required to assess its generalizability beyond the selected botanical drugs.

### 3.6 Effects of botanical drugs on intestinal histomorphology in mice with ceftriaxone-induced gut microbiota dysbiosis

**FIGURE 8:**
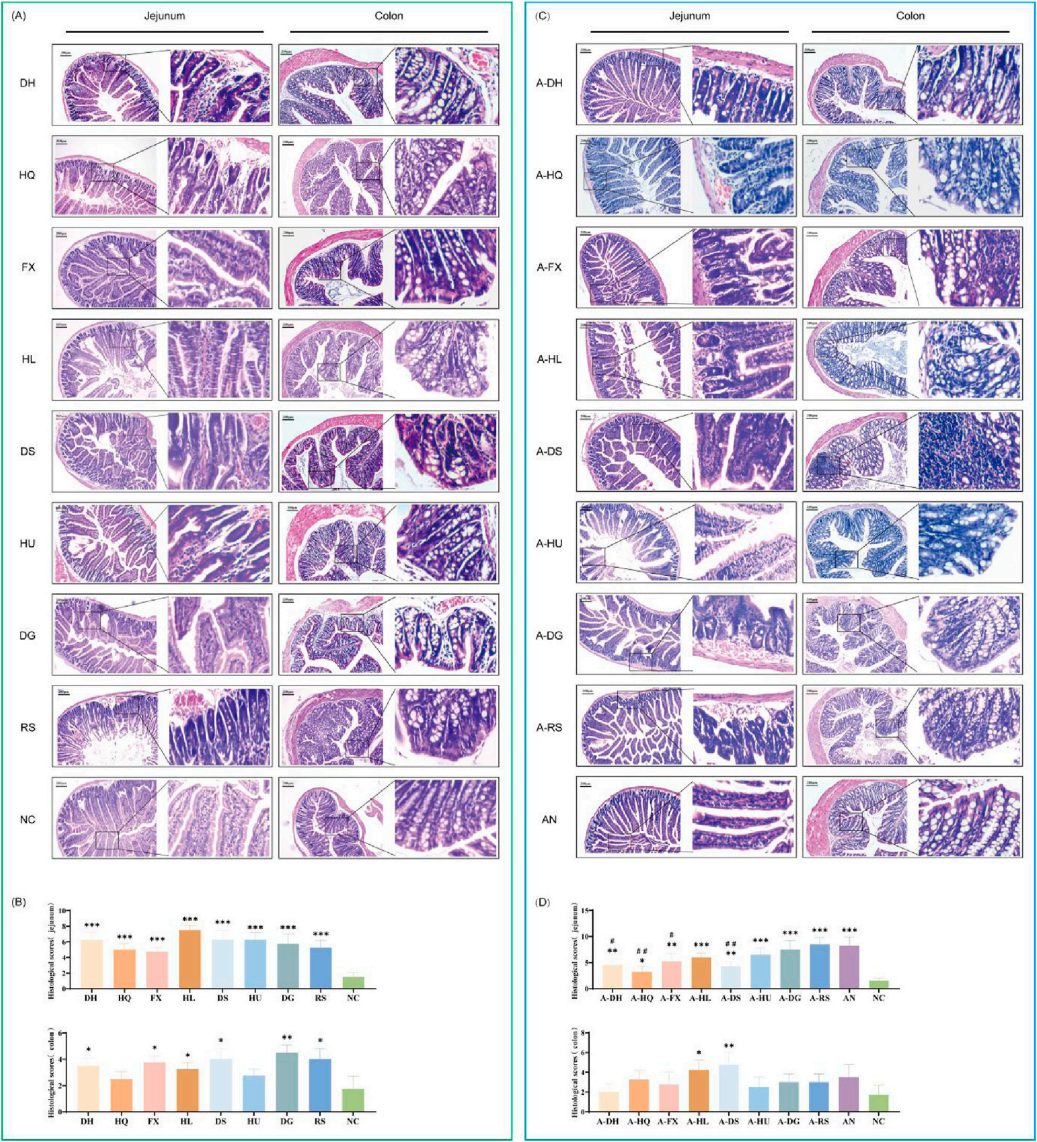
Effects of Various Botanical Drugs on the Structure of Mouse Intestinal Tissue: **(A)** Morphology of jejunal and colonic tissue under normal conditions. **(B)** Histopathological scores of the jejunum and colon under normal conditions. **(C)** Morphology of jejunal and colonic tissue in the context of gut microbiota dysbiosis. **(D)** Histopathological scores of the jejunum and colon in the context of gut microbiota dysbiosis. Compared with the NC group, * indicates *P* < 0.05, ** indicates *P* < 0.01, and *** indicates *P* < 0.001. Compared to the AN group, # indicates *P* < 0.05, and ## indicates *P* < 0.01.

The structural morphology of the intestinal epithelium was examined in mice treated with eight botanical drugs selected based on TCM cold-hot classifications. The results revealed that, under normal conditions, both cold- and hot-classified botanical drugs exhibited varying degrees of pathological alterations in the structure of small intestinal epithelial cells. These alterations were manifested as disrupted villus architecture, increased cell shedding, abnormal hyperplasia of small intestinal glands, and infiltration of inflammatory cells. Particularly, the histological changes in the small intestine were more pronounced in the HL and HU groups. Upon examining the colonic tissues across all groups, we found localized loss, shrinkage, and shedding of colonic mucosal epithelium, disorganization of glandular structures, decreased numbers of goblet cells, and infiltration of inflammatory cells within the mucosal layer in the HQ, HL, DS, HU, DG, and RS groups. Notably, hot-classified drugs induced more pronounced alterations in the colonic tissue architecture compared to cold botanical drugs (Figures 8A,B).

In the natural recovery group with microbiota dysbiosis (AN group), we observed a significant reduction in the components of small intestinal epithelial cells, accompanied by necrosis, vacuolar degeneration of enterocytes, and infiltration of inflammatory cells around the cells. Among the botanical drugs intervention groups, the A-RS, A-DG, and A-HU groups demonstrated the most prominent changes in small intestinal histology. Hot-classified drugs exhibited a stronger destructive effect on the small intestinal structure of mice with gut microbiota dysbiosis compared to cold-classified drugs. All experimental groups exhibited pathological changes in colonic mucosal epithelial cell structure, characterized by localized loss, shrinkage, and shedding, along with disorganization of glandular structures, decreased goblet cell numbers, and increased inflammatory cells within the mucosal layer. Notably, the colonic muscularis propria was significantly thinner in all botanical drugs groups compared to the NC and AN groups (Figures 8C,D).

### 3.7 Effects of botanical drugs on key indicators in mice with ceftriaxone-induced gut microbiota dysbiosis

**FIGURE 9:**
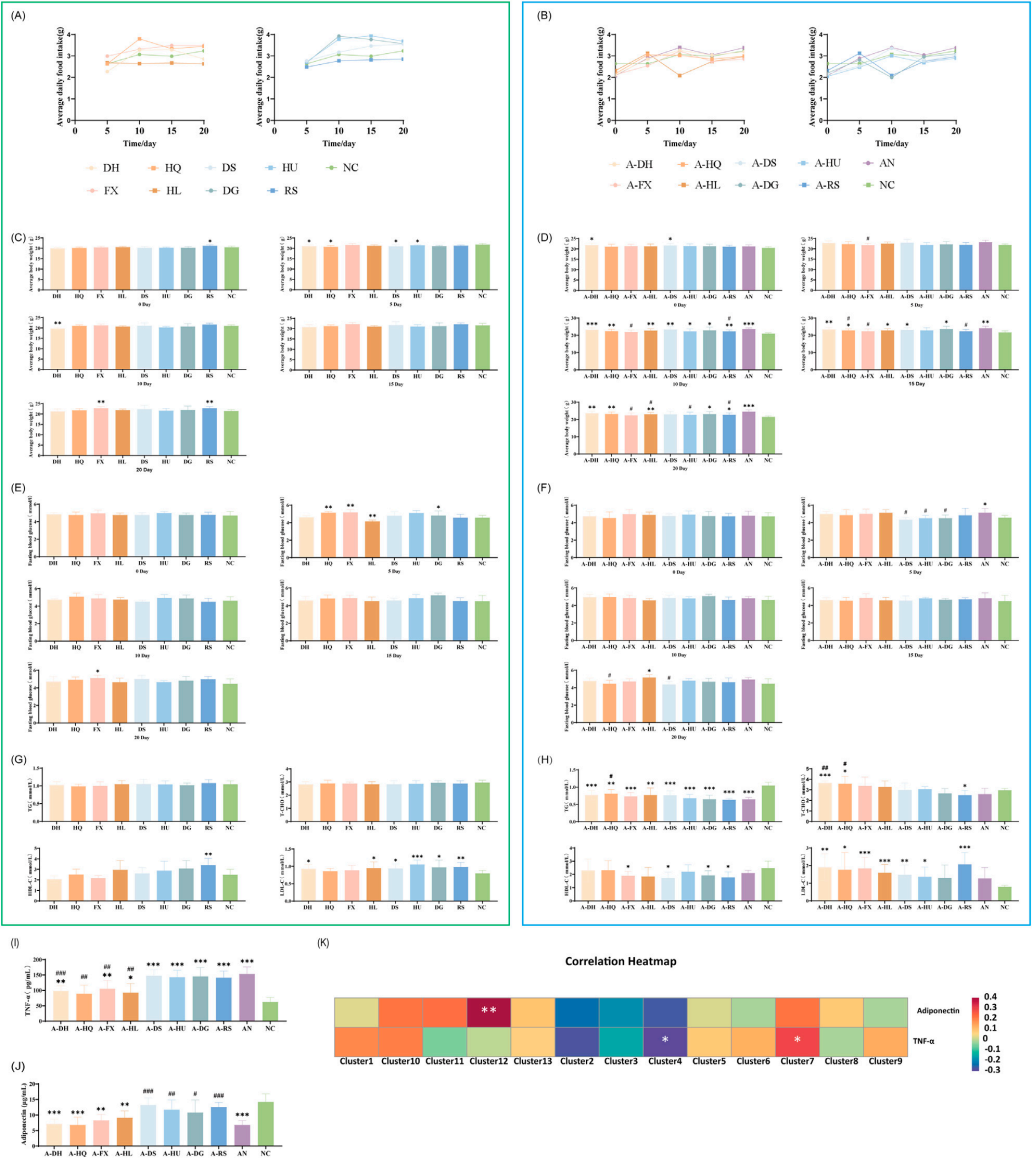
Effects of Various Botanical Drugs on Physiological Indices, Glucose and Lipid Metabolism, and Inflammatory/Metabolic Markers in Mice: **(A)** Mean daily intake under normal conditions. **(B)** Mean daily intake in the context of gut microbiota dysbiosis. **(C)** Body weight under normal conditions. **(D)** Body weight in the context of gut microbiota dysbiosis. **(E)** Fasting blood glucose levels at different time points under normal conditions. **(F)** Fasting blood glucose levels at different time points in the context of gut microbiota dysbiosis. **(G)** Serum concentrations of triglycerides (TG), total cholesterol (T-CHO), high-density lipoprotein cholesterol (HDL-C), and low-density lipoprotein cholesterol (LDL-C) under normal conditions. **(H)** Serum concentrations of TG, T-CHO, HDL-C, and LDL-C in the context of gut microbiota dysbiosis. **(I)** Serum TNF-α concentrations in the context of gut microbiota dysbiosis. **(J)** Serum adiponectin concentrations in the context of gut microbiota dysbiosis. **(K)** Correlation Analysis Between Gut Microbiota and Inflammatory/Metabolic Markers. Statistical analyses were performed using unpaired Student's t-tests. Comparisons with the NC group are indicated by * for *P* < 0.05, ** for *P* < 0.01, and *** for *P* < 0.001. Comparisons with the AN group are indicated by # for *P* < 0.05, ## for *P* < 0.01 and ### for *P* < 0.001.

The study investigated dietary intake and body weight changes in mice treated with eight botanical drugs. In normal mice, botanical drug administration induced a transient reduction in food intake during the first 5 days, followed by gradual recovery to baseline levels by day 20 (Figure 9A). In contrast, ceftriaxone-treated mice with gut microbiota dysbiosis (AN group) exhibited similar food intake trends post-botanical drug intervention, though significant variations were observed in the A-HL and A-DG groups compared to the AN group (Figure 9B).

Body weight changes: After the administration of botanical drugs, normal mice experienced a transient decrease in body weight followed by a gradual increase, remaining consistently below the NC group (Figure 9C). In mice with gut microbiota dysbiosis treated with botanical drugs, the cold-classified drug groups showed a slow increase in body weight, while the hot-classified drug groups experienced a slight decrease in body weight towards the end of the experiment (except for the A-RS group) (Figure 9D).

### 3.8 Differential modulation of glucose and lipid metabolism by botanical drugs classified as cold or hot in TCM

Assessment of blood glucose and lipid levels in mice treated with eight botanical drugs showed that both cold- and hot-classified botanical drugs maintained relatively stable fasting blood glucose levels in normal mice (Figure 9E). In the ceftriaxone-induced gut microbiota dysbiosis model, cold-classified drugs tended to stabilize or slightly elevate blood glucose levels, while hot-classified drugs caused a slight decrease in fasting blood glucose levels within 5 days, followed by stabilization (*P* < 0.05, except for the A-RS group) (Figure 9F).

After 20 days of botanical drugs intervention in normal mice, there were no significant differences in serum TG and TC concentrations compared to the NC group. The HDL-C concentration was significantly higher in the RS group than in the NC group (*P* < 0.01), while LDL-C concentrations were significantly elevated in the DH (*P* < 0.05), HL (*P* < 0.05), DS (*P* < 0.05), HU (*P* < 0.001), DG (*P* < 0.05), and RS (*P* < 0.01) groups compared to the NC group. Both cold- and hot-classified botanical drugs did not exhibit significant differences in regulating serum lipids in normal mice (Figure 9G). In mice with ceftriaxone-induced gut microbiota dysbiosis, after 20 days of natural recovery (AN group), serum TG concentrations remained significantly lower than those in the NC group (*P* < 0.001), while other lipid indicators showed no significant differences. Cold- and hot-classified botanical drugs did not differ significantly in regulating serum TG and HDL-C concentrations, but hot-classified drugs showed stronger effects on regulating serum TC and LDL-C levels compared to cold-classified drugs (Figure 9H).

### 3.9 Correlation between microbial clusters and inflammatory/metabolic markers

To elucidate the anti-inflammatory and metabolic regulatory properties of botanical drugs, serum levels of TNF-α and adiponectin were analyzed in a gut microbiota dysbiosis model across the experimental groups. Additionally, correlation analyses were conducted between these biomarkers and microbial clusters.

#### 3.9.1 TNF-α levels

Cold-classified drugs demonstrated a general trend toward reducing TNF-α levels, although the statistical significance varied (Figure 9I)

Cold-classified drugs: *Rheum palmatum* L.: 98.5 ± 16.2 pg/mL (compared to AN: 153.4 ± 22.9 pg/mL, *P* < 0.001); *Scutellaria baicalensis* Georgi: 89.3 ± 27.5 pg/mL (*P* < 0.01); *Senna alexandrina* Mill.: 105.1 ± 28.0 pg/mL (*P* < 0.01); *Coptis chinensis* Franch.: 92.6 ± 29.1 pg/mL (*P* < 0.01).

No significant reduction in TNF-α levels was observed with the following hot-classified drugs: *Codonopsis pilosula* (Franch.) Nannf. at 147.2 ± 19.7 pg/mL; *Astragalus membranaceus* (Fisch.) Bunge at 142.8 ± 22.3 pg/mL; *Angelica sinensis* (Oliv.) Diels at 145.5 ± 28.8 pg/mL; and *Panax ginseng* C.A. Mey. at 140.9 ± 21.7 pg/mL.

#### 3.9.2 Adiponectin levels

Hot-classified drugs exhibited a significant capacity to increase adiponectin levels, although there was variability among the different drugs (Figure 9J).

Cold-classified drugs have a minimal impact on adiponectin levels. The following concentrations were observed: *Rheum palmatum* L. at 7.1 ± 1.6 μg/mL; *Scutellaria baicalensis* Georgi at 6.8 ± 2.5 μg/mL; *Senna alexandrina* Mill. at 8.3 ± 1.9 μg/mL; and *Coptis chinensis* Franch. at 9.1 ± 2.2 μg/mL.

Hot-classified drugs: *Codonopsis pilosula* (Franch.) Nannf.: 13.2 ± 2.3 μg/mL (compared to AN: 6.8 ± 1.4 μg/mL, *P* < 0.001); *Astragalus membranaceus* (Fisch.) Bunge: 11.7 ± 3.1 μg/mL (*P* < 0.01); *Angelica sinensis* (Oliv.) Diels: 10.8 ± 4.0 μg/mL (*P* < 0.05); *Panax ginseng* C.A. Mey.: 12.5 ± 1.5 μg/mL (*P* < 0.001).

Spearman correlation analysis (Figure 9K) indicated that: TNF-α levels negatively correlated with Cluster 4 (*Allobaculum*, *Bifidobacterium*; *P* < 0.05), a taxon enriched in cold-classified drug groups. Adiponectin levels negatively correlated with Cluster 12 (*Clostridia*; *P* < 0.01), a taxon promoted by hot-classified drugs. These associations suggest a potential microbiota-mediated mechanism for the observed metabolic and inflammatory outcomes.

## 4 Discussion

Single botanical drugs, classified by their distinct properties of four natures (cold, hot, warm, cool), five flavors, and meridian tropism, play a crucial role in TCM prescriptions aimed at achieving therapeutic objectives such as “eliminating excess and supplementing deficiency” (Xu et al., 2019). Our study systematically investigated eight pharmacopeia-compliant botanical drugs (*Rheum palmatum* L., *Scutellaria baicalensis* Georgi, etc.) to explore how TCM’s emic classification of “cold” and “hot” properties differentially modulates gut microbiota dysbiosis. By integrating phytochemical profiling with longitudinal microbiota analysis, we demonstrate that both cold- and hot-classified drugs universally stabilize dominant gut genera while selectively enriching low-abundance taxa associated with anti-inflammatory or metabolic functions. These findings provide a microbial perspective to decode TCM’s empirical classifications, bridging traditional knowledge with modern microbiota science.

### 4.1 Phytochemical basis of TCM cold-hot properties and microbial interactions

In the selected botanical drugs, cold-classified drugs (e.g., *Coptis chinensis* Franch.) exhibited enrichment of alkaloids (e.g., berberine, OB = 36.86%) and monoterpenes, compounds with documented anti-inflammatory properties (Guo et al., 2021; Yu et al., 2022). Conversely, hot-classified drugs (e.g., *Panax ginseng* C.A. Mey.) demonstrated higher levels of saponins (e.g., ginsenoside-Rh4, OB = 31.11%) and phenylpropanoids, which are implicated in lipid metabolism (Lee et al., 2021; Li et al., 2024). These phytochemical distinctions, though observed within a limited drug cohort, align with our microbiota findings: cold-classified drugs enriched *Akkermansia* and *Bifidobacterium* (anti-inflammatory taxa), while hot-classified drugs promoted *Clostridia* and *Eubacterium coprostanoligenes* (metabolic-modulating genera) (Cani et al., 2022; Kenny et al., 2020). For instance, *Akkermansia* has been linked to immune regulation, through phospholipid-induced cytokine modulation (Bae et al., 2022); whereas *Clostridia* correlates with cholesterol catabolism via IsmA gene-mediated pathways (Li et al., 2024). This functional alignment supports TCM’s emic framework—rooted in empirical observations of therapeutic effects—and highlights its potential relevance to microbial ecology. However, the generalizability of such chemical-microbial associations requires validation across expanded drug cohorts, given the inherent diversity within TCM classifications (e.g., Angelica sinensis contains fewer saponins than Panax ginseng).

### 4.2 Taxon-specific modulation and host physiology

Both cold- and hot-classified drugs restored dominant genera (*Lactobacillus*, *Muribaculaceae*) and normalized alpha diversity by day 20. However, their differential enrichment of low-abundance taxa (<10% relative abundance) significantly influenced host physiology.

Cold-classified drugs preferentially enriched anti-inflammatory taxa, with the abundance of *Akkermansia* in the cold-classified drug-treated dysbiosis groups (A-DH: 628.6, A-HQ: 583, A-FX: 476, A-HL: 663.8) significantly exceeding that of the AN group (112.2) and the NC group (0) (Cani et al., 2022). Concurrently, *Bifidobacterium* levels in the cold-classified drug groups (A-DH: 428, A-HQ: 583, A-FX: 476, A-HL: 663.8) surpassed both the AN (112.2) and NC (372.6) baselines (Sanders et al., 2019). These enrichments correlated with reduced serum TNF-α levels (*P* < 0.01 vs. AN group), potentially mediated by *Bifidobacterium*-derived indole-3-acetic acid (IAA), which suppresses hepatic inflammation through aryl hydrocarbon receptor activation (Yong et al., 2024). Mechanistically, *Akkermansia*-secreted phospholipid A15:0-i15:0 PE further modulated immune activation by stimulating TNF-α and IL-6 secretion (Bae et al., 2022), aligning with the anti-inflammatory effects traditionally attributed to TCM cold-classified drugs.

In contrast, hot-classified drugs significantly increased the abundance of metabolic-modulating genera. The levels of *Clostridia* (A-DS: 280.8, A-HU: 226.2, A-DG: 225.0, A-RS: 440.4) and *Eubacterium coprostanoligenes* (A-DS: 42.8, A-HU: 40.2, A-DG: 33.2, A-RS: 88.8) in hot-classified drug-treated dysbiosis models markedly exceeded those in the AN (184.2, 66.0) and NC (61.0, 11.0) groups. This taxon-specific enrichment facilitated cholesterol catabolism through *Clostridia*’s sugar alcohol metabolism (Tiffany et al., 2021) and IsmA gene-mediated pathways (Li et al., 2024), leading to significant reductions in serum TC and LDL-C (Kenny et al., 2020). These metabolic improvements resonate with the TCM concept of “warming the middle, the direct causal relationship requires further validation.

Notably, the inverse correlation between TNF-α and adiponectin levels (*P* < 0.05) highlights the complementary roles of cold- and hot-classified drugs in modulating inflammation and metabolism.

### 4.3 Inflammatory and metabolic cross-talk: dynamics of TNF-α and adiponectin

The interplay between TNF-α and adiponectin further underscores the dual regulatory roles of cold- and hot-classified botanical drugs. The suppression of TNF-α by cold-classified drugs (e.g., *Rheum palmatum* L.: 98.5 ± 16.2 pg/mL vs. AN: 153.4 ± 22.9 pg/mL) aligns with their traditional anti-inflammatory indications. This effect may be linked to *Akkermansia* enrichment, which has been associated with IL-6 modulation in prior studies (Bae et al., 2022). In contrast, hot-classified drugs significantly elevated adiponectin levels (e.g., *Codonopsis pilosula* (Franch.) Nannf.: 13.2 ± 2.3 μg/mL vs. AN: 6.8 ± 1.4 μg/mL), a crucial adipokine that enhances insulin sensitivity and counteracts metabolic dysfunction (Petersen and Shulman, 2018). This inverse relationship between TNF-α and adiponectin highlights their antagonistic roles in inflammation and metabolism. While cold-classified drugs may target inflammatory pathways as TCM theory suggests, hot-classified drugs appear to enhance metabolic homeostasis, illustrating a complementary mechanism that warrants further exploration.

### 4.4 Intestinal barrier and physiological correlations

Notably, cold-classified drugs induced mild inflammatory cell infiltration in intestinal tissues, possibly reflecting immune activation through *Akkermansia*-mediated IL-22 pathways (Liu et al., 2024), though causal relationships require validation via germ-free models. Conversely, hot-classified drugs caused structural alterations, such as reduced villus height, which may represent a trade-off for their metabolic-enhancing effects. Despite these transient disruptions, both types of drugs promoted epithelial repair by day 20, as evidenced by histopathological recovery. This observation parallels findings in colitis models where TCM-classified drugs restored mucosal integrity through microbiota-dependent mechanisms (Li et al., 2022), suggesting shared repair pathways across traditional and biomedical frameworks.

### 4.5 Implications of the emic-etic framework in TCM research

The emic-etic framework, first proposed by Pike (1967) and later applied to ethnomedicine by Headland et al. (1992), distinguishes between culture-specific (“insider”) and universal (“outsider”) perspectives. In TCM, the emic classification of “cold” and “hot” properties reflects empirical observations of therapeutic effects, while an etic approach would prioritize biochemical metrics. In this study, the TCM emic classification—based on centuries of observational practice (where cold-classified drugs inhibit metabolism and exhibit anti-inflammatory effects, while hot-classified drugs enhance local blood supply, and promote metabolism)—gained mechanistic support through data on microbiota-host interactions. For example, the enrichment of *Akkermansia* and the reduction in serum TNF-α levels by cold-classified drugs validate their traditional anti-inflammatory indications, whereas the promotion of *Clostridia* and the improvement in serum adiponectin levels by hot-classified drugs align with metabolic enhancement. This synergy between emic classifications and microbial biomarkers presents a novel pathway for modernizing TCM research; however, further validation in chronic disease models is necessary to assess broader applicability.

### 4.6 Limitations and future directions

This study has several limitations that warrant attention. First, the selection of only eight TCM-classified botanical drugs—while compliant with pharmacopeial standards—limits the representativeness of the cold-hot categories. For instance, the exclusion of hot-natured Zingiber officinale (ginger), which is rich in non-saponin compounds like gingerols, highlights the need for broader investigations that incorporate additional drugs, such as *Atractylodes macrocephala* Koidz. (known for “spleen invigoration to validate the generalizability of our findings. Second, while the ceftriaxone-induced dysbiosis model is clinically relevant, it may not fully replicate chronic disease-associated microbiota-host interactions. Third, the chemical screening criteria (e.g., OB ≥ 30%) prioritized systemic bioavailability but potentially overlooked locally active metabolites critical to TCM’s empirical efficacy, such as gut-targeted phytochemicals with low absorption. Finally, the mechanistic links (e.g., *Akkermansia*-mediated IL-22 pathways) and causal relationships between microbial taxa and host outcomes require validation using germ-free models or metabolite tracing.

## 5 Conclusion

Botanical drugs classified as cold or hot in TCM stabilize the diversity and hierarchy of dominant gut microbiota (those with over 10% abundance) universally, while differentially modulating low-abundance taxa (those with less than 10% abundance). This taxon-specific enrichment corresponds with their traditional anti-inflammatory (cold) and metabolic-enhancing (hot) properties, as outlined in TCM theory. Although *Akkermansia* and *Clostridia* may serve as provisional microbial indicators for further investigation, their generalizability necessitates validation across a broader range of drug cohorts. These findings connect TCM’s empirical framework with microbiota science, providing a mechanistic foundation to reinterpret traditional classifications through the lens of microbial ecology.

## Data Availability

The datasets presented in this study can be found in online repositories. The names of the repository/repositories and accession number(s) can be found in the article/Supplementary Material.

## Abbreviations

TC, Total Cholesterol; TG, Triglyceride; HDL-C, High-Density Lipoprotein Cholesterol; LDL-C, Low-Density Lipoprotein Cholesterol; TCMSP, The Traditional Chinese Medicine Systems Pharmacology Database and Analysis Platform; OB, Oral Bioavailability; DL, Drug-likeness; HL, Half-life; SPF, Specific-Pathogen Free; TCM, Traditional Chinese Medicine; PCoA, Principal Coordinate Analysis.
