# Supplementary material for: Cold- and hot-classified botanical drugs differentially modulate gut microbiota: linking TCM emic classification to microbial ecology
Source: Front Pharmacol. 2025 May 16;16:1545619. doi: 10.3389/fphar.2025.1545619 (PMC12122528; doi:10.3389/fphar.2025.1545619)
Supplement: Supplementary file 1 [file Supplementaryfile1.docx]

**Cold- and Hot-Classified Botanical Drugs Differentially Modulate Gut Microbiota: Linking TCM Emic Classification to Microbial Ecology**

Huan Yang ^1^, Chenyi Shao ^1^, Zhihao Liu ^1^, Xiaoyu Zhang ^1^, Yinhui Liu ^1^, Jing Xiao^2, *^ , Li Tang ^1, *^

1 Department of Microecology, College of Basic Medical Sciences, Dalian Medical University, Dalian, 116044, China

2 Department of Oral Pathology, College of Stomatology, Dalian Medical University, Dalian, 116044, China

* Correspondence author:

Prof. Xiao Jing

E-**mail:** [xiaoj@dmu.edu.cn](mailto:xiaoj@dmu.edu.cn)

Prof. Li Tang

1. **mail:** [tangl@dmu.edu.cn](mailto:tangl@dmu.edu.cn)

Funding : This research was funded by Dalian Medical University Interdisciplinary Research Cooperation Project Team Funding (JCHZ2023003) and the National Natural Science Foundation of China (grant number no. 81970719).

*Supplementary S1*


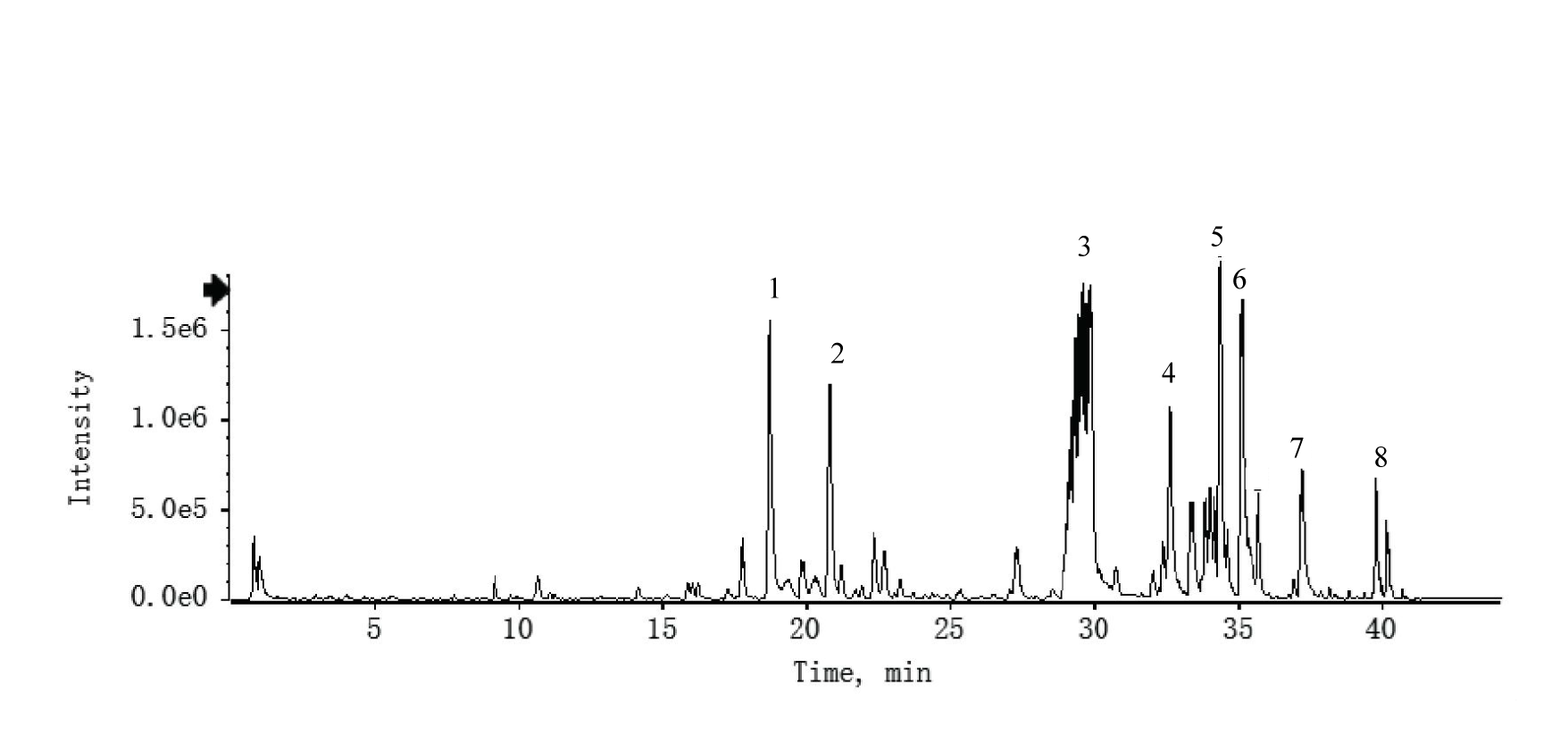


**Figures S1** Chromatograms of the Chemical Profiles in Botanical Drugs.

Table S1. Chemical Profile Information of Botanical Drugs Based on UPLC−Q-TOF/MS

| No | RT（min） | ESI | calcd | obsed | ppm | formula | components | MS/MS | peak area | From |
| --- | --- | --- | --- | --- | --- | --- | --- | --- | --- | --- |
| 1 | 18.71 | [M-H]^-^ | 547.1492 | 547.1457 | 6.4 | C_20_H_18_ClNO | Berberine | 487.1292;457.1177;367.0849;337.0740 | 67284024 | *Coptis chinensis* Franch. |
| 2 | 20.78 | [M-H]^-^ | 547.1488 | 547.1457 | 5.6 | C_15_H_10_O_5_ | Aloeemodin | 547.1485;487.1267;457.1157;427.1051;367.0824;337.0715 | 57259001 | *Rheum palmatum* L. |
| 3 | 29.65 | [M-H]^-^ | 445.0798 | 445.0776 | 4.9 | C₂₀H₂₈O₈ | Lobetyolin | 445.1212;269.0470;239.0385 | 68440739 | *Codonopsis pilosula* (Franch.) Nannf. |
| 4 | 32.61 | [M-H]^-^ | 445.0803 | 445.0776 | 8.7 | C_42_H_38_O_20_ | Sennoside | 445.1164;269.0468;239.0355;113.0245 | 45267445 | *Senna alexandrina* Mill. |
| 5 | 34.37 | [M-H]^-^ | 459.0966 | 459.0933 | 7.2 | C_41_H_68_O_14_ | Astragaloside A | 299.0555;283.0612;268.0385;239.0366;211.0402 | 40123887 | *Astragalus membranaceus* (Fisch.) Bunge |
| 6 | 35.13 | [M-H]^-^ | 459.0968 | 459.0933 | 7.7 | C_54_H_92_O_23_ | Ginsenoside Rb1 | 459.0963;283.0617'268.0385;175.0250;113.0247 | 77473526 | *Panax ginseng* C.A.Mey |
| 7 | 37.21 | [M-H]^-^ | 269.0471 | 269.0455 | 5.8 | C_21_H_18_O_11_ | Baicalin | 269.0478;241.0517;225.0553;197.061;195.0463;136.9892 | 40766175 | *Scutellaria baicalensis* Georgi |
| 8 | 39.81 | [M+H]^+^ | 285.0759 | 285.0758 | 0.5 | C_10_H_10_O_4_ | Ferulic Acid | 269.0440;253.0492;225.0542;213.0544;197.059 | 27166851 | *Angelica sinensis* (Oliv.) Diels |

Table S2 Screening of the Active Ingredients of Botanical Drugs

| Name | Chinese Name | Ingredients | OB（％） | DL | HL |
| --- | --- | --- | --- | --- | --- |
| *Rheum palmatum* L. | Dahuang | Sennoside D_qt | 61.06 | 0.61 | 33.92 |
|  |  | Sennoside E_qt | 50.69 | 0.61 | 33.6 |
|  |  | palmidin A | 32.45 | 0.65 | 32.14 |
|  |  | rhein | 47.07 | 0.28 | 32.12 |
|  |  | aloe-emodin | 83.38 | 0.24 | 31.49 |
|  |  | Emodin-1-O-beta-D-glucopyranoside | 44.81 | 0.8 | 29.79 |
|  |  | Physciondiglucoside | 41.65 | 0.63 | 27.61 |
|  |  | Torachrysone-8-O-beta-D-(6'-oxayl)-glucoside | 43.02 | 0.74 | 16.29 |
|  |  | Mutatochrome | 48.64 | 0.61 | 15.73 |
|  |  | EUPATIN | 50.8 | 0.41 | 13.94 |
|  |  | Daucosterol_qt | 35.89 | 0.7 | 6.12 |
|  |  | Procyanidin B-5,3'-O-gallate | 31.99 | 0.32 | 5.98 |
|  |  | beta-sitosterol | 36.91 | 0.75 | 5.36 |
| *Scutellaria baicalensis* Georgi | Huangqin | acacetin | 34.97 | 0.24 | 17.25 |
|  |  | wogonin | 30.68 | 0.23 | 17.75 |
|  |  | (2R)-7-hydroxy-5-methoxy-2-phenylchroman-4-one | 55.23 | 0.2 | 17.02 |
|  |  | baicalein | 33.52 | 0.21 | 16.25 |
|  |  | 5,8,2'-Trihydroxy-7-methoxyflavone | 37.01 | 0.27 | 16.17 |
|  |  | 5,7,2,5-tetrahydroxy-8,6-dimethoxyflavone | 33.82 | 0.45 | 15.94 |
|  |  | Carthamidin | 41.15 | 0.24 | 15.81 |
|  |  | 2,6,2',4'-tetrahydroxy-6'-methoxychaleone | 69.04 | 0.22 | 21.89 |
|  |  | Dihydrobaicalin_qt | 40.04 | 0.21 | 16.13 |
|  |  | Eriodyctiol (flavanone) | 41.35 | 0.24 | 15.88 |
|  |  | Salvigenin | 49.07 | 0.33 | 15.87 |
|  |  | 5,2',6'-Trihydroxy-7,8-dimethoxyflavone | 45.05 | 0.33 | 16.37 |
|  |  | 5,7,2',6'-Tetrahydroxyflavone | 37.01 | 0.24 | 18 |
|  |  | dihydrooroxylin A | 38.72 | 0.23 | 17.58 |
|  |  | Skullcapflavone II | 69.51 | 0.44 | 16.14 |
|  |  | oroxylin a | 41.37 | 0.23 | 17.15 |
|  |  | Panicolin | 76.26 | 0.29 | 16.78 |
|  |  | 5,7,4'-Trihydroxy-8-methoxyflavone | 36.56 | 0.27 | 16.93 |
|  |  | NEOBAICALEIN | 104.34 | 0.44 | 16.5 |
|  |  | DIHYDROOROXYLIN | 66.06 | 0.23 | 17.17 |
|  |  | beta-sitosterol | 36.91 | 0.75 | 5.36 |
|  |  | sitosterol | 36.91 | 0.75 | 5.37 |
|  |  | Norwogonin | 39.4 | 0.21 | 16.93 |
|  |  | 5,2'-Dihydroxy-6,7,8-trimethoxyflavone | 31.71 | 0.35 | 16.47 |
|  |  | Stigmasterol | 43.83 | 0.76 | 5.57 |
|  |  | coptisine | 30.67 | 0.86 | 9.33 |
|  |  | epiberberine | 43.09 | 0.78 | 6.1 |
|  |  | Moslosooflavone | 44.09 | 0.25 | 17.02 |
|  |  | 11,13-Eicosadienoic acid, methyl ester | 39.28 | 0.23 | 5.44 |
|  |  | 5,7,4'-trihydroxy-6-methoxyflavanone | 36.63 | 0.27 | 16.12 |
|  |  | 5,7,4'-trihydroxy-8-methoxyflavanone | 74.24 | 0.26 | 16.85 |
|  |  | rivularin | 37.94 | 0.37 | 16.25 |
| *Senna* *alexandrina* Mill. | Fanxieye | Physciondiglucoside | 41.65 | 0.63 | 27.61 |
|  |  | rhein | 47.07 | 0.28 | 32.12 |
|  |  | Sennoside E_qt | 50.69 | 0.61 | 33.6 |
|  |  | Emodin-1-O-beta-D-glucopyranoside | 44.81 | 0.8 | 29.79 |
|  |  | Sennoside D_qt | 61.06 | 0.61 | 33.92 |
|  |  | Dihydroxydianthrone | 74.55 | 0.57 | 33.57 |
|  |  | sitosterol | 36.91 | 0.75 | 5.37 |
|  |  | kaempferol | 41.88 | 0.24 | 14.74 |
|  |  | Stigmasterol | 43.83 | 0.76 | 5.57 |
| *Coptis chinensis* Franch. | Huanglian | berberine | 36.86 | 0.78 | 6.57 |
|  |  | berberrubine | 35.74 | 0.73 | 6.46 |
|  |  | epiberberine | 43.09 | 0.78 | 6.1 |
|  |  | (R)-Canadine | 55.37 | 0.77 | 6.41 |
|  |  | Berlambine | 36.68 | 0.82 | 7.33 |
|  |  | Corchoroside A_qt | 104.95 | 0.78 | 6.68 |
|  |  | Palmidin A | 35.36 | 0.65 | 33.17 |
|  |  | quercetin | 46.43 | 0.28 | 14.4 |
|  |  | coptisine | 30.67 | 0.86 | 9.33 |
|  |  | Worenine | 45.83 | 0.87 | 8.41 |
| *Codonopsis pilosula* (Franch.) Nannf. | Danshen | poriferasta-7,22E-dien-3beta-ol | 42.98 | 0.76 | 5.48 |
|  |  | Perlolyrine | 65.95 | 0.27 | 12.62 |
|  |  | ZINC03978781 | 43.83 | 0.76 | 5.79 |
|  |  | Stigmasterol | 43.83 | 0.76 | 5.57 |
|  |  | 7-Methoxy-2-methyl isoflavone | 42.56 | 0.2 | 16.89 |
|  |  | Spinasterol | 42.98 | 0.76 | 5.32 |
|  |  | Chrysanthemaxanthin | 38.72 | 0.58 | 17.47 |
|  |  | Frutinone A | 65.9 | 0.34 | 19.1 |
|  |  | luteolin | 36.16 | 0.25 | 15.94 |
|  |  | stigmast-7-enol | 37.42 | 0.75 | 6.28 |
|  |  | 3-beta-Hydroxymethyllenetanshiquinone | 32.16 | 0.41 | 22.51 |
|  |  | methyl icosa-11,14-dienoate | 39.67 | 0.23 | 5.24 |
|  |  | 5alpha-Stigmastan-3,6-dione | 33.12 | 0.79 | 5.19 |
|  |  | 7-(beta-Xylosyl)cephalomannine_qt | 38.33 | 0.29 | 5.97 |
|  |  | Daturilin | 50.37 | 0.77 | 5.73 |
|  |  | glycitein | 50.48 | 0.24 | 16.32 |
|  |  | Spinoside A | 39.97 | 0.4 | 8.24 |
| *Astragalus membranaceus* (Fisch.) Bunge | Huangqi | Mairin | 55.38 | 0.78 | 8.87 |
|  |  | Jaranol | 50.83 | 0.29 | 15.5 |
|  |  | hederagenin | 36.91 | 0.75 | 5.35 |
|  |  | (3S,8S,9S,10R,13R,14S,17R)-10,13-dimethyl-17-[(2R,5S)-5-propan-2-yloctan-2-yl]-2,3,4,7,8,9,11,12,14,15,16,17-dodecahydro-1H-cyclopenta[a]phenanthren-3-ol | 36.23 | 0.78 | 5.22 |
|  |  | isorhamnetin | 49.6 | 0.31 | 14.34 |
|  |  | 3,9-di-O-methylnissolin | 53.74 | 0.48 | 9 |
|  |  | 9,10-dimethoxypterocarpan-3-O-尾-D-glucoside | 36.74 | 0.92 | 13.06 |
|  |  | (6aR,11aR)-9,10-dimethoxy-6a,11a-dihydro-6H-benzofurano[3,2-c]chromen-3-ol | 64.26 | 0.42 | 8.49 |
|  |  | Bifendate | 31.1 | 0.67 | 17.96 |
|  |  | formononetin | 69.67 | 0.21 | 17.04 |
|  |  | isoflavanone | 109.99 | 0.3 | 15.51 |
|  |  | Calycosin | 47.75 | 0.24 | 17.1 |
|  |  | kaempferol | 41.88 | 0.24 | 14.74 |
|  |  | FA | 68.96 | 0.71 | 24.81 |
|  |  | 1,7-Dihydroxy-3,9-dimethoxy pterocarpene | 39.05 | 0.48 | 7.95 |
|  |  | quercetin | 46.43 | 0.28 | 14.4 |
| *Angelica sinensis* (Oliv.)Diels | Danggui | beta-sitosterol | 36.91 | 0.75 | 5.36 |
|  |  | Stigmasterol | 43.83 | 0.76 | 5.57 |
| *Panax Ginseng* C. A. Mey | Renshen | Stigmasterol | 43.83 | 0.76 | 5.57 |
|  |  | beta-sitosterol | 36.91 | 0.75 | 5.36 |
|  |  | Inermin | 65.83 | 0.54 | 11.73 |
|  |  | kaempferol | 41.88 | 0.24 | 14.74 |
|  |  | Chrysanthemaxanthin | 38.72 | 0.58 | 17.47 |
|  |  | Celabenzine | 101.88 | 0.49 | 8.15 |
|  |  | Deoxyharringtonine | 39.27 | 0.81 | 7.9 |
|  |  | Dianthramine | 40.45 | 0.2 | 5.14 |
|  |  | arachidonate | 45.57 | 0.2 | 7.56 |
|  |  | Frutinone A | 65.9 | 0.34 | 19.1 |
|  |  | ginsenoside rh2 | 36.32 | 0.56 | 11.08 |
|  |  | Ginsenoside-Rh4_qt | 31.11 | 0.78 | 6.97 |
|  |  | Girinimbin | 61.22 | 0.31 | 8.17 |
|  |  | Gomisin B | 31.99 | 0.83 | 7.81 |
|  |  | malkangunin | 57.71 | 0.63 | 4.09 |
|  |  | Panaxadiol | 33.09 | 0.79 | 6.34 |
|  |  | suchilactone | 57.52 | 0.56 | 9.03 |
|  |  | alexandrin_qt | 36.91 | 0.75 | 5.53 |
|  |  | ginsenoside Rg5_qt | 39.56 | 0.79 | 5.65 |
|  |  | Fumarine | 59.26 | 0.83 | 23.46 |

*Supplementary S2*

*
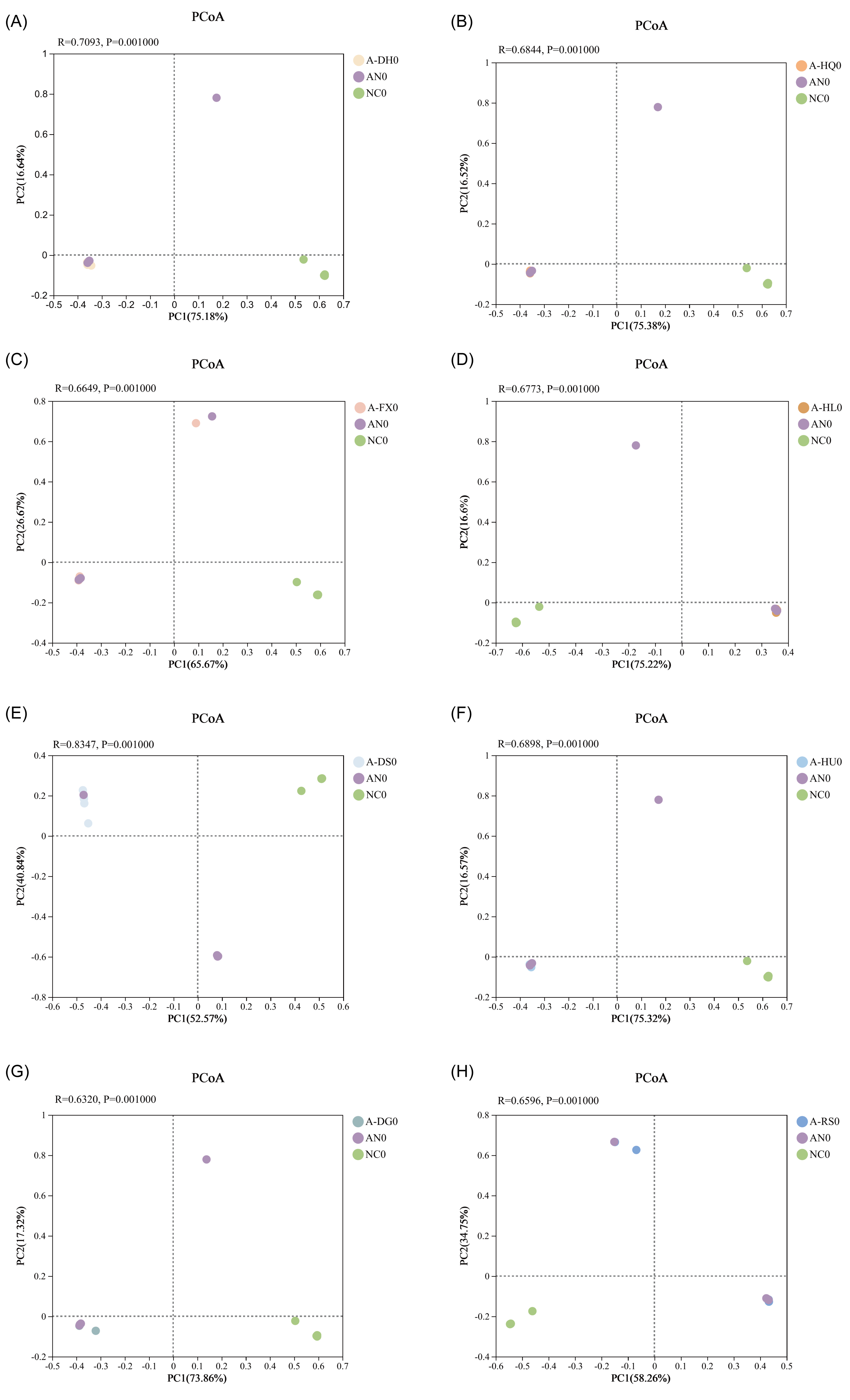
*

**Figures S2** PCoA plot of a mouse model with disturbed gut microbiota from ceftriaxone sodium: (A) A-DH Group, (B) A-HQ Group, (C) A-FX Group, (D) A-HL Group, (E) A-DS Group, (F) A-HU Group, (G) A-DG Group, (H) A-RS Group.

*Supplementary S3*


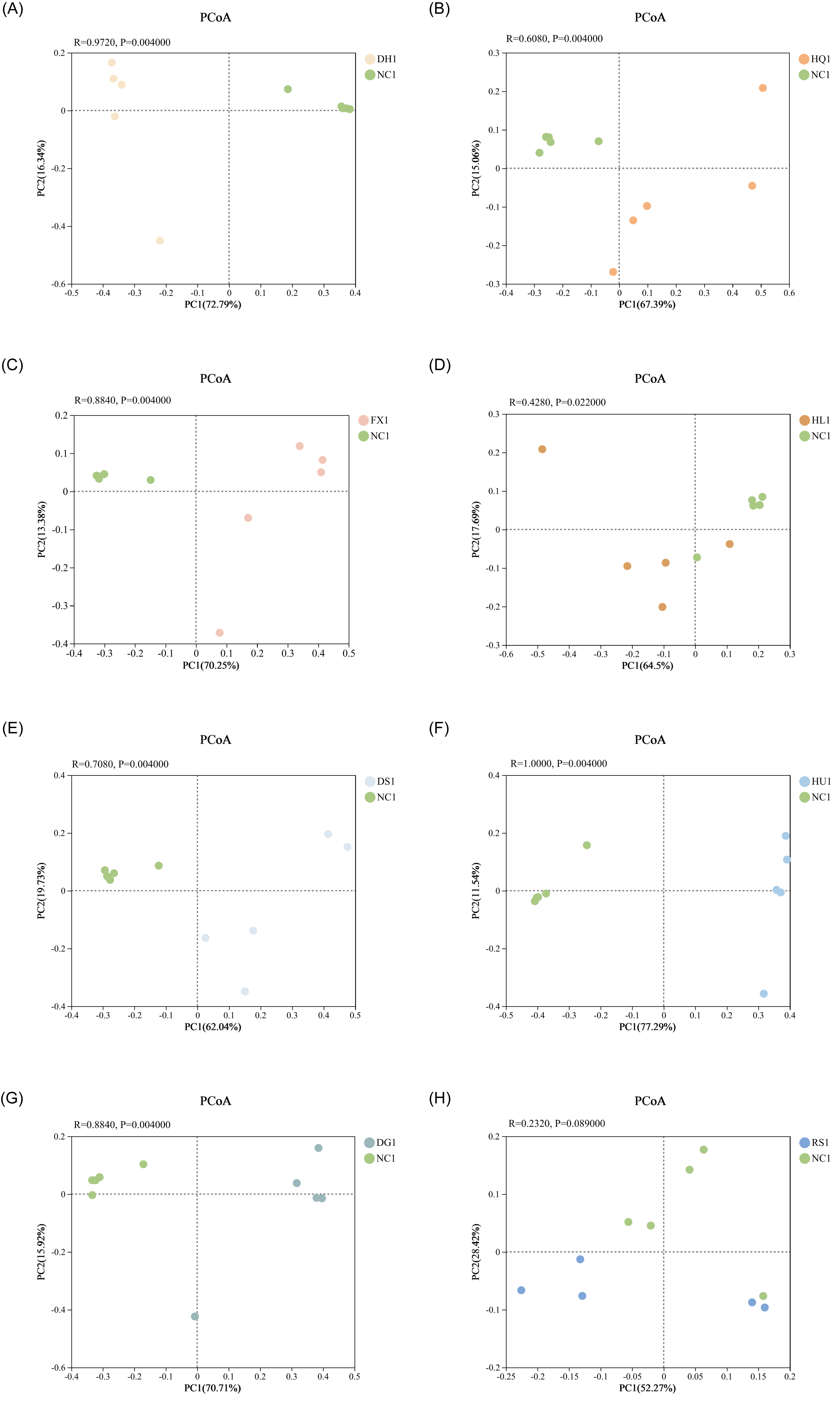


**Figures S3** PCoA plot shows the impact of a 5-day botanical drug intervention on mice gut microbiota under normal conditions: (A) DH Group, (B) HQ Group, (C) FX Group, (D) HL Group, (E) DS Group, (F) HU Group, (G) DG Group, (H) RS Group.

*Supplementary S4*


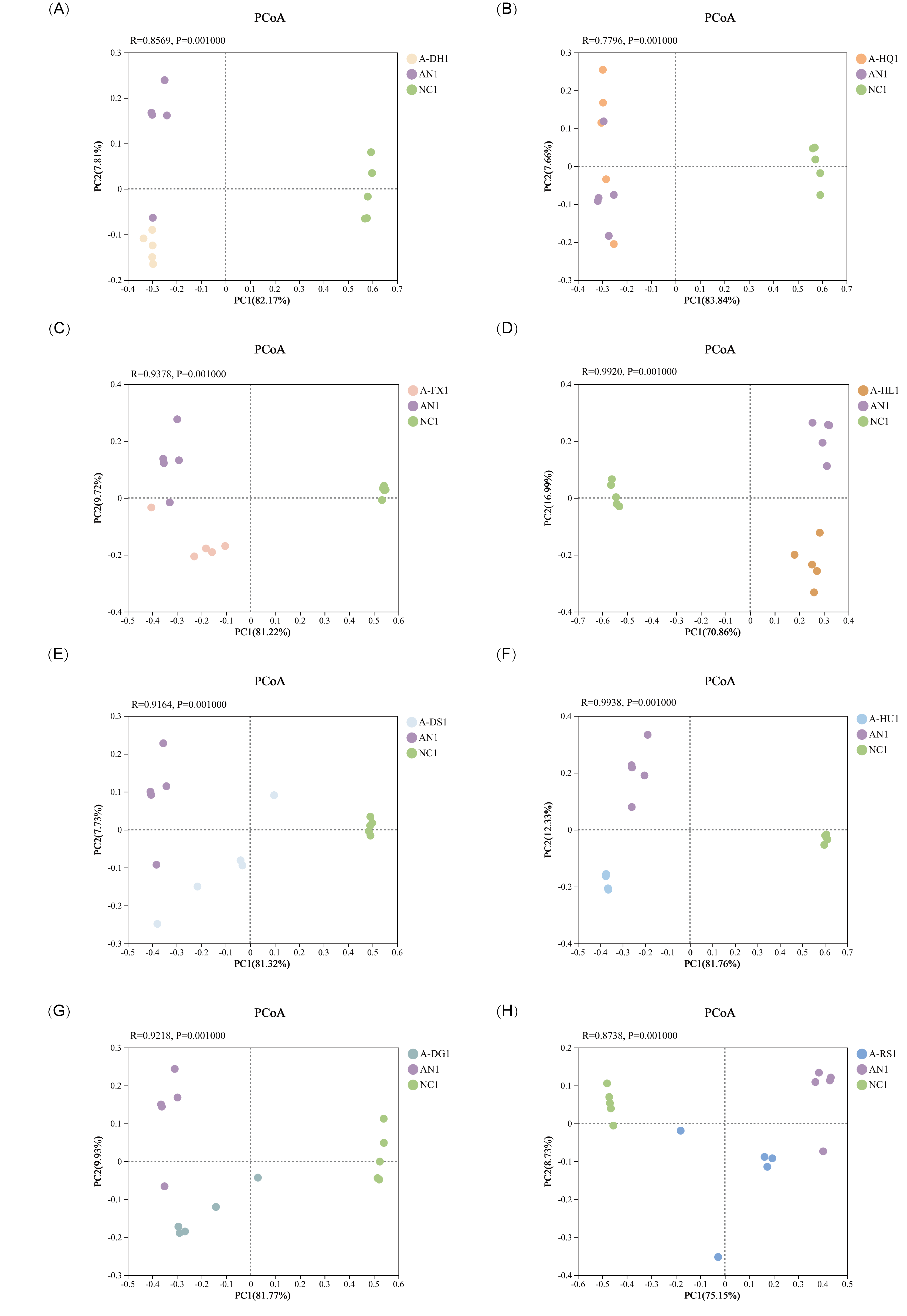


**Figures S4** PCoA plot illustrates the effects of a 5-day botanical drug intervention on the gut microbiota of mice in the context of gut microbiota dysbiosis: (A) A-DH Group, (B) A-HQ Group, (C) A-FX Group, (D) A-HL Group, (E) A-DS Group, (F) A-HU Group, (G) A-DG Group, (H) A-RS Group.

*Supplementary S5*


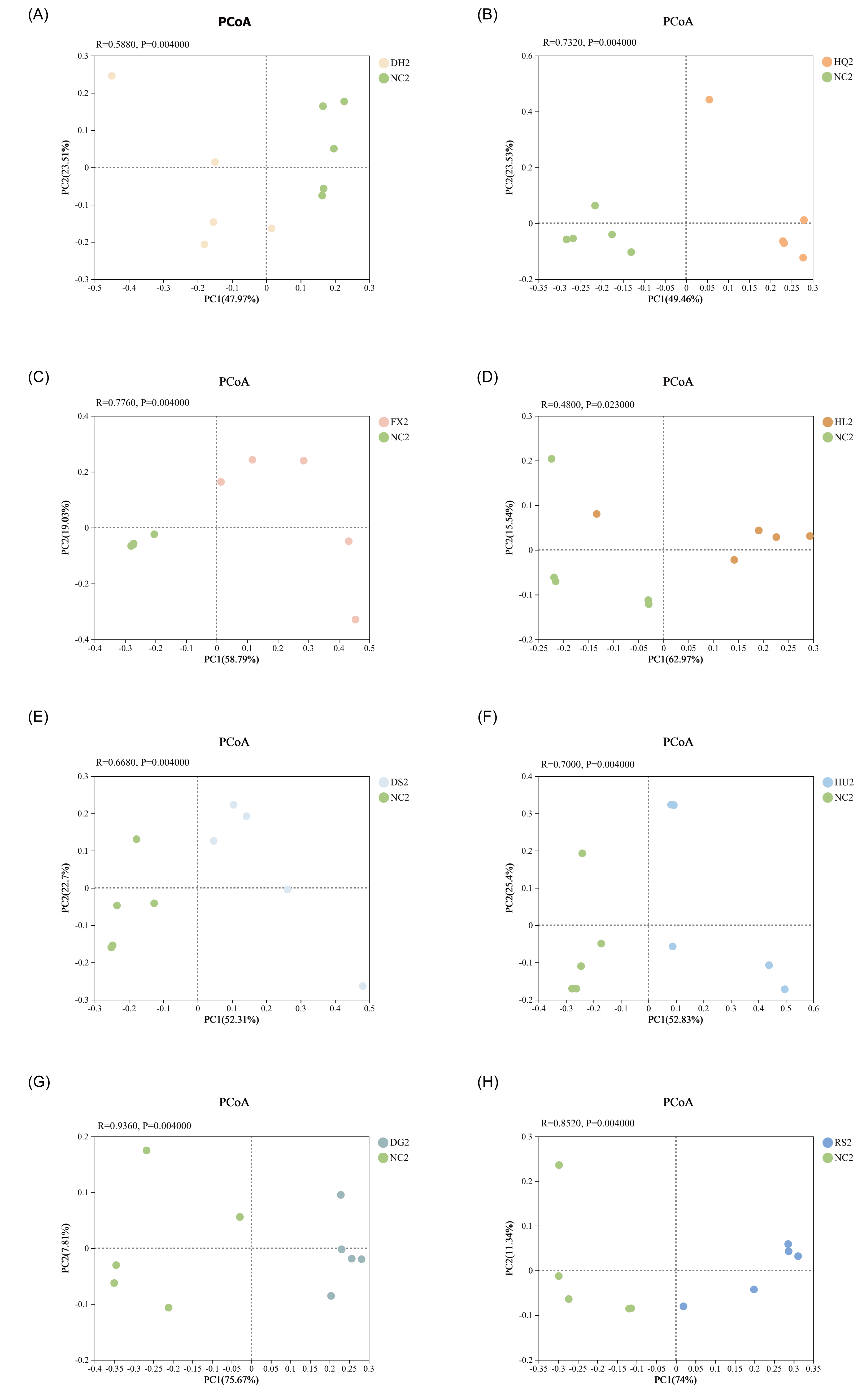


**Figures S5** PCoA plot shows the impact of a 10-day botanical drug intervention on mice gut microbiota under normal conditions: (A) DH Group, (B) HQ Group, (C) FX Group, (D) HL Group, (E) DS Group, (F) HU Group, (G) DG Group, (H) RS Group.

*Supplementary S6*


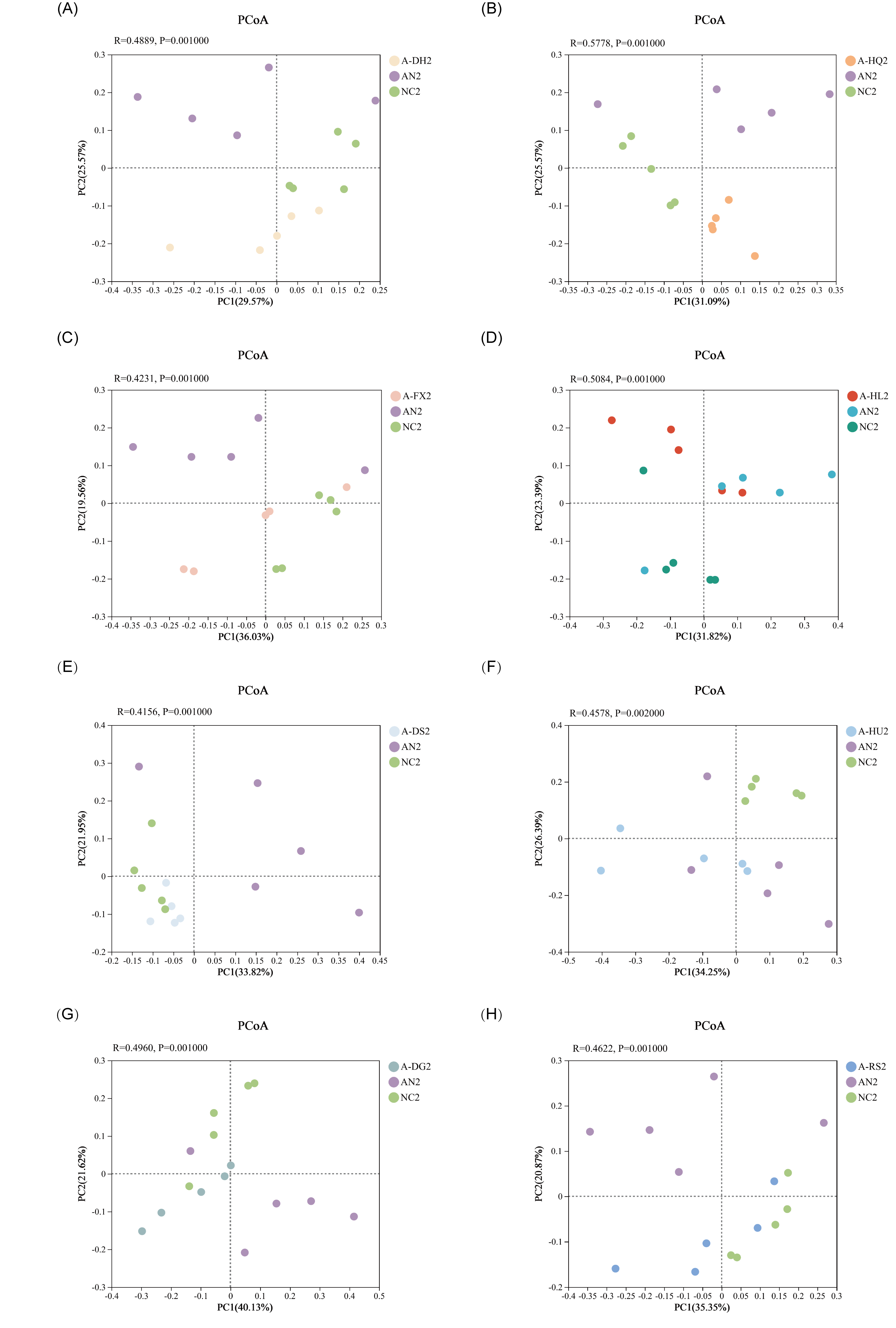


**Figures S6** PCoA plot illustrates the effects of a 10-day botanical drug intervention on the gut microbiota of mice in the context of gut microbiota dysbiosis: (A) A-DH Group, (B) A-HQ Group, (C) A-FX Group, (D) A-HL Group, (E) A-DS Group, (F) A-HU Group, (G) A-DG Group, (H) A-RS Group.

*Supplementary S7*


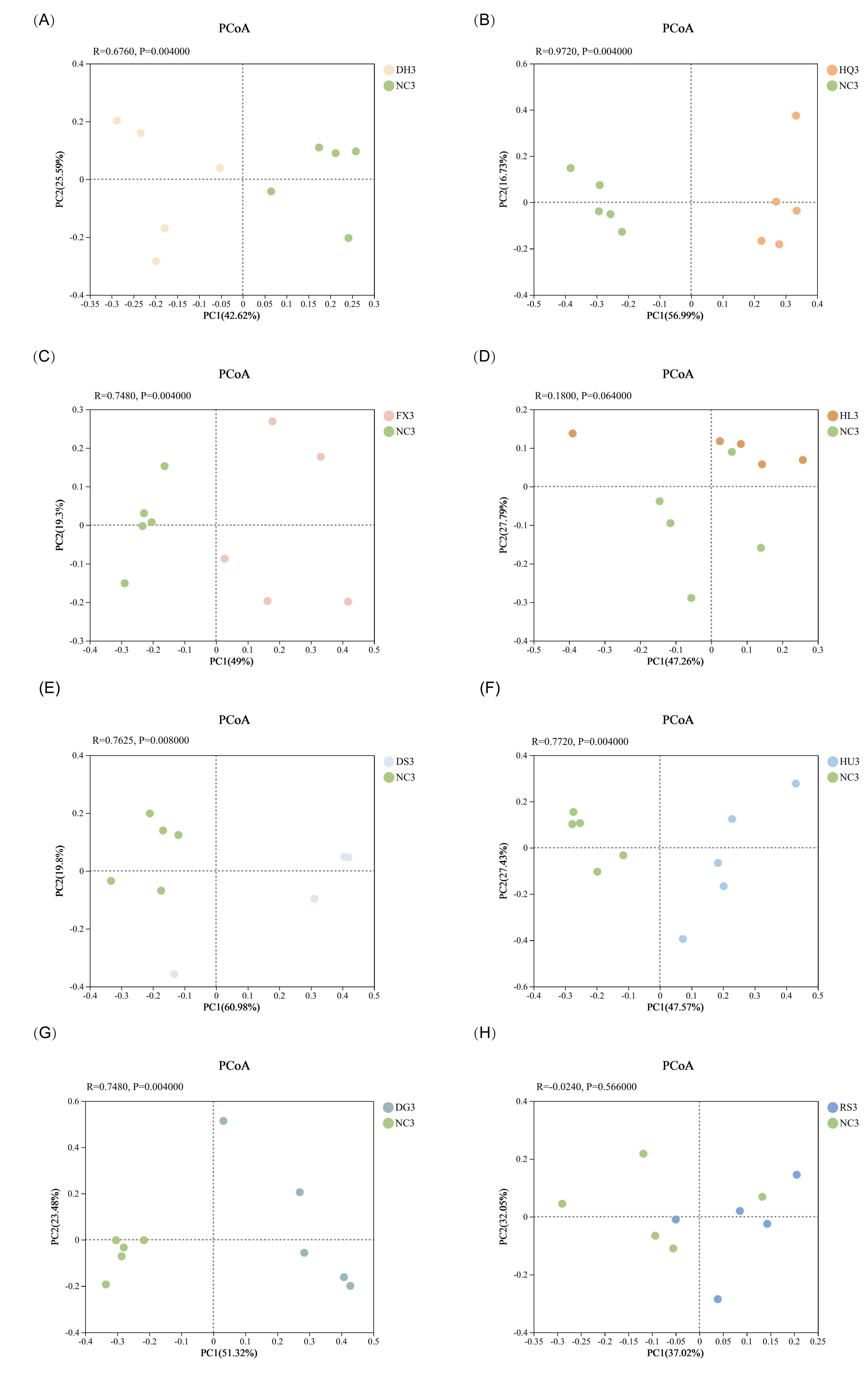


**Figures S7** PCoA plot shows the impact of a 15-day botanical drug intervention on mice gut microbiota under normal conditions: (A) DH Group, (B) HQ Group, (C) FX Group, (D) HL Group, (E) DS Group, (F) HU Group, (G) DG Group, (H) RS Group.

*Supplementary S8*


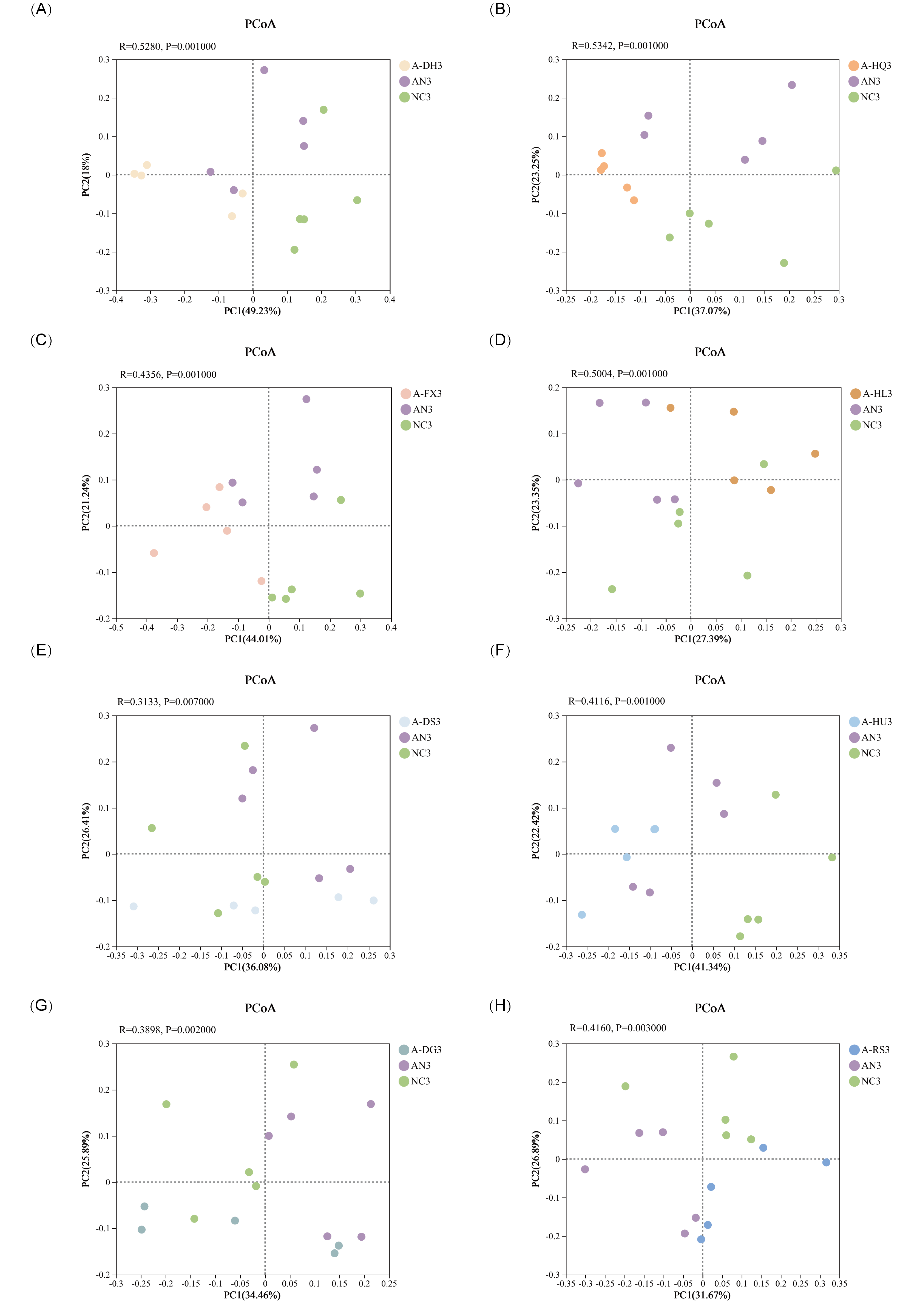


**Figures S8** PCoA plot illustrates the effects of a 15-day botanical drug intervention on the gut microbiota of mice in the context of gut microbiota dysbiosis: (A) A-DH Group, (B) A-HQ Group, (C) A-FX Group, (D) A-HL Group, (E) A-DS Group, (F) A-HU Group, (G) A-DG Group, (H) A-RS Group.

*Supplementary S9*


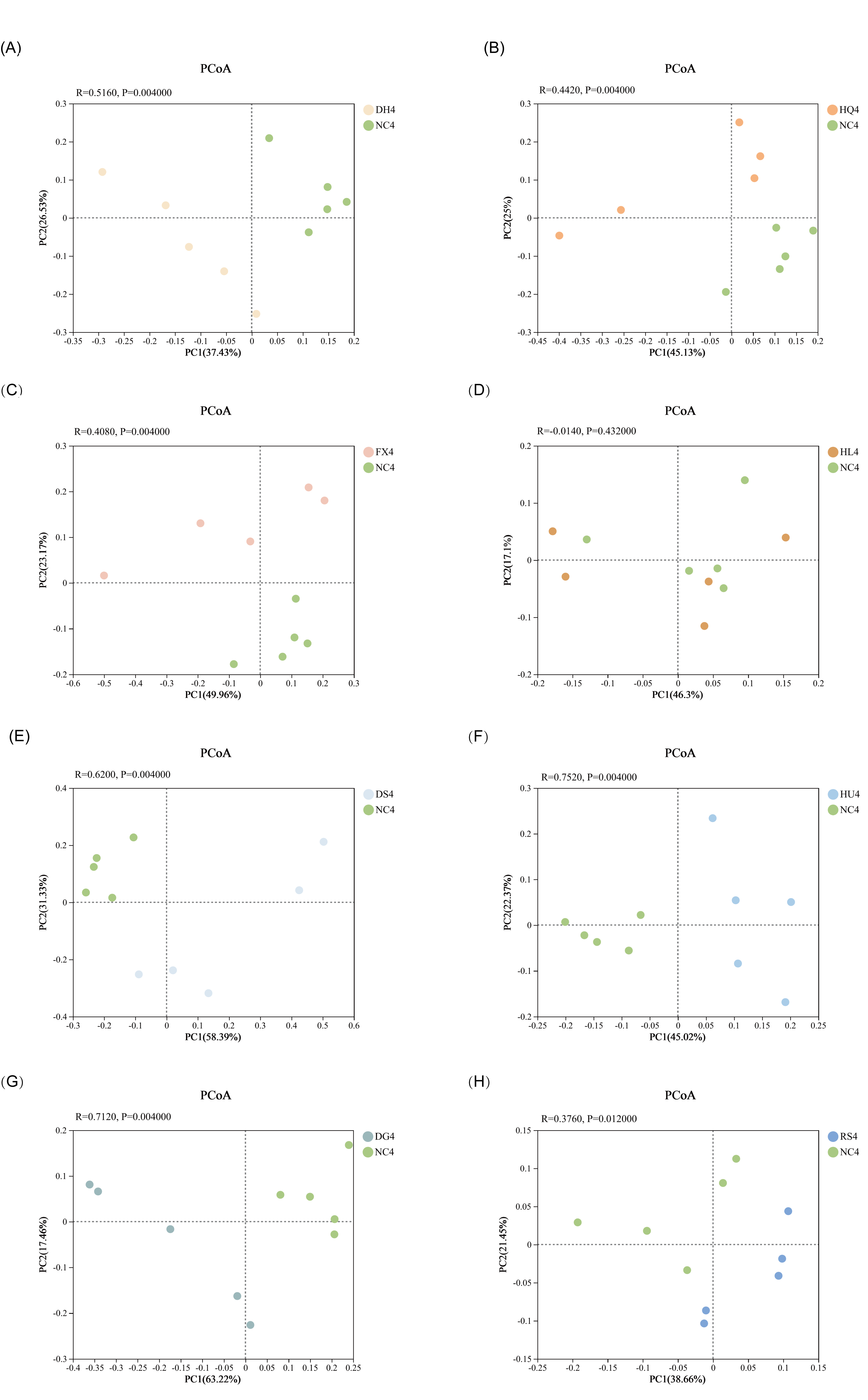


**Figures S9** PCoA plot shows the impact of a 20-day botanical drug intervention on mice gut microbiota under normal conditions: (A) DH Group, (B) HQ Group, (C) FX Group, (D) HL Group, (E) DS Group, (F) HU Group, (G) DG Group, (H) RS Group.

*Supplementary S10*


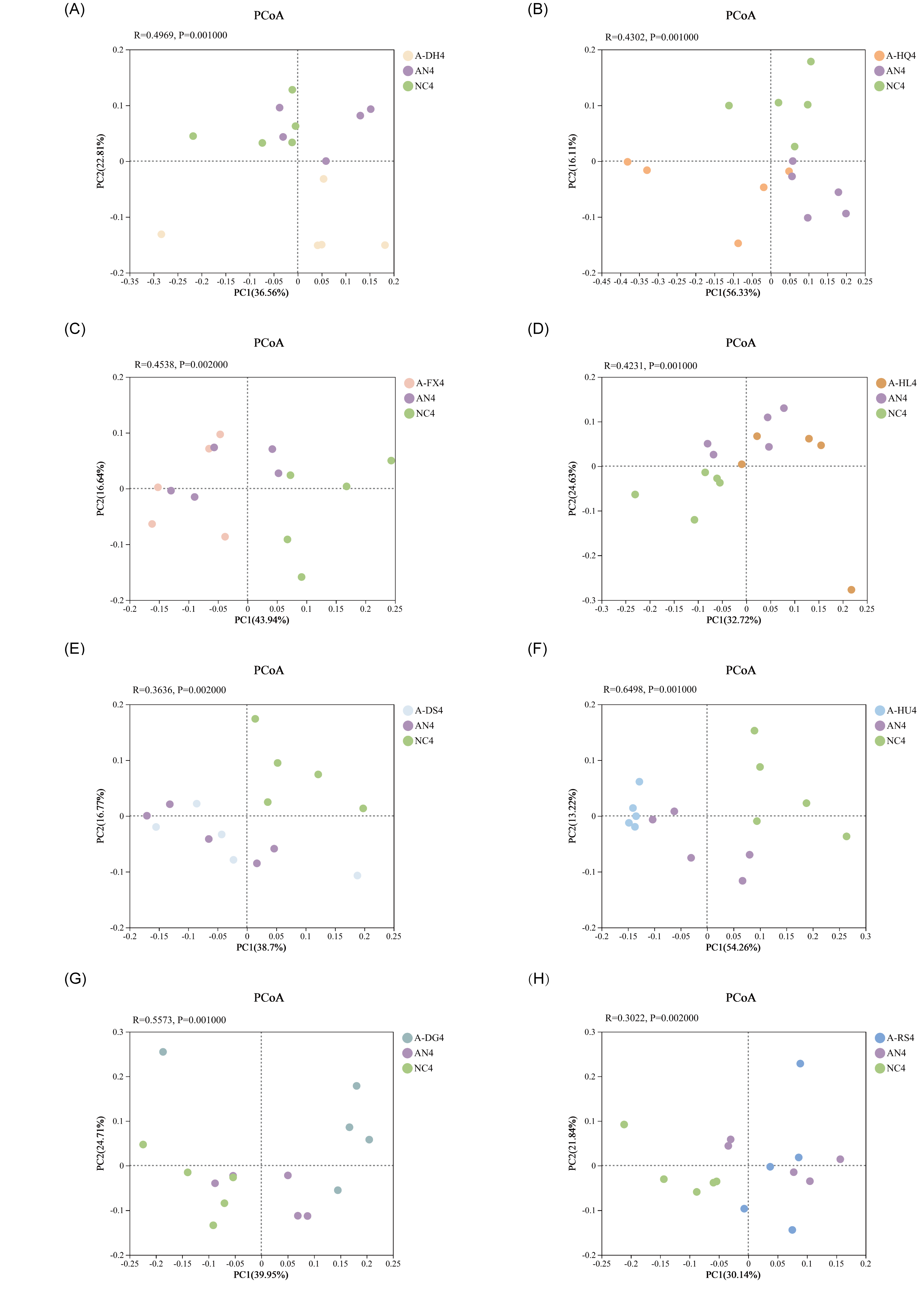


**Figures S10** PCoA plot illustrates the effects of a 20-day botanical drug intervention on the gut microbiota of mice in the context of gut microbiota dysbiosis: (A) A-DH Group, (B) A-HQ Group, (C) A-FX Group, (D) A-HL Group, (E) A-DS Group, (F) A-HU Group, (G) A-DG Group, (H) A-RS Group.

*Supplementary S11*


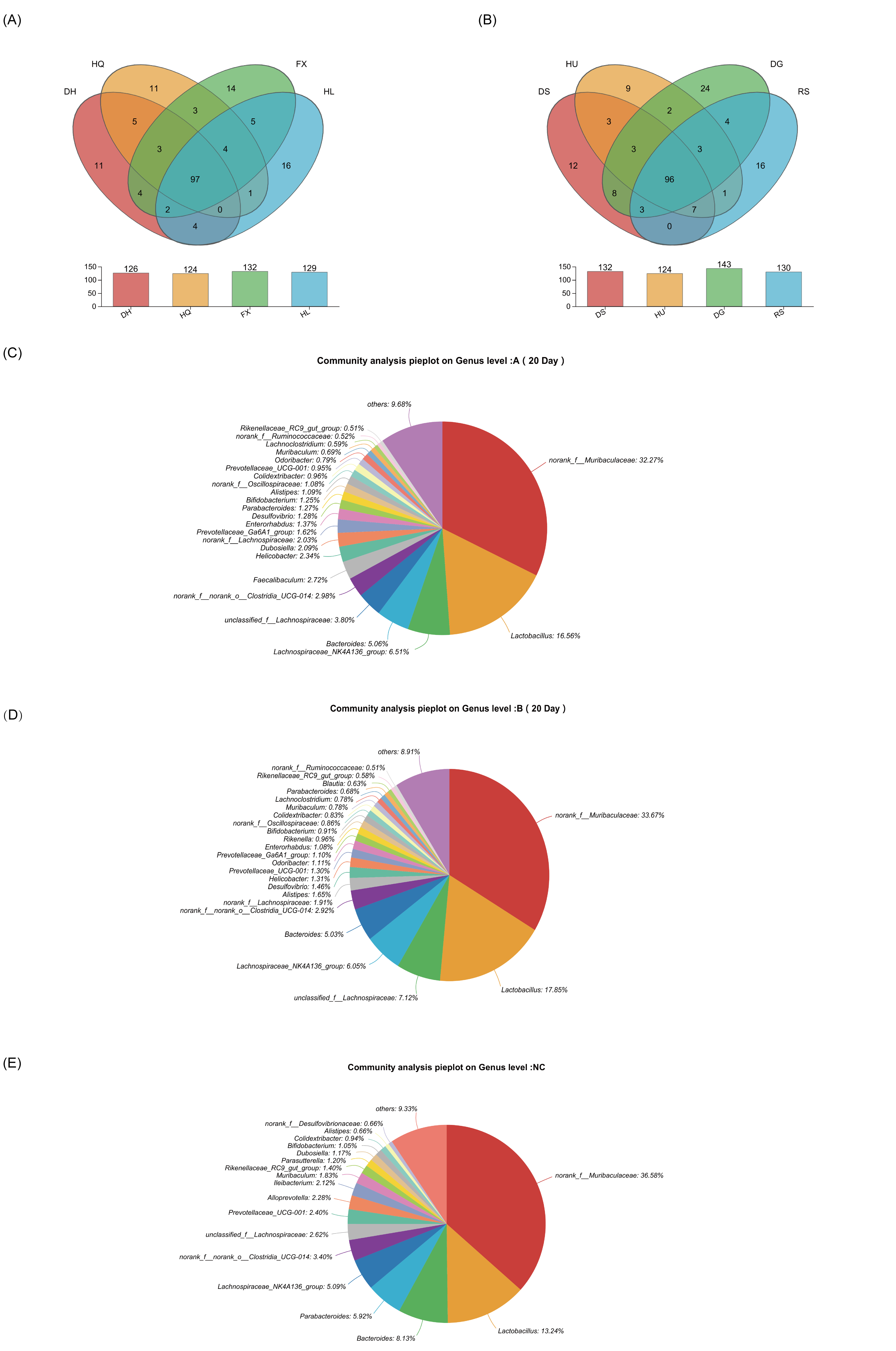


**Figures S11** The effects of traditional Chinese medicines on mouse gut microbiota after 20 days of intervention are shown under normal conditions. (A) Venn diagram of genus-level composition in cold-classified drug groups. (B) Venn diagram of genus-level composition in hot-classified drug groups. (C) Gut microflora structure in the cold-classified drug group. (D) Gut microflora structure in the hot-classified drug group. (E) Gut microbiota structure in the NC group.

*Supplementary S12*


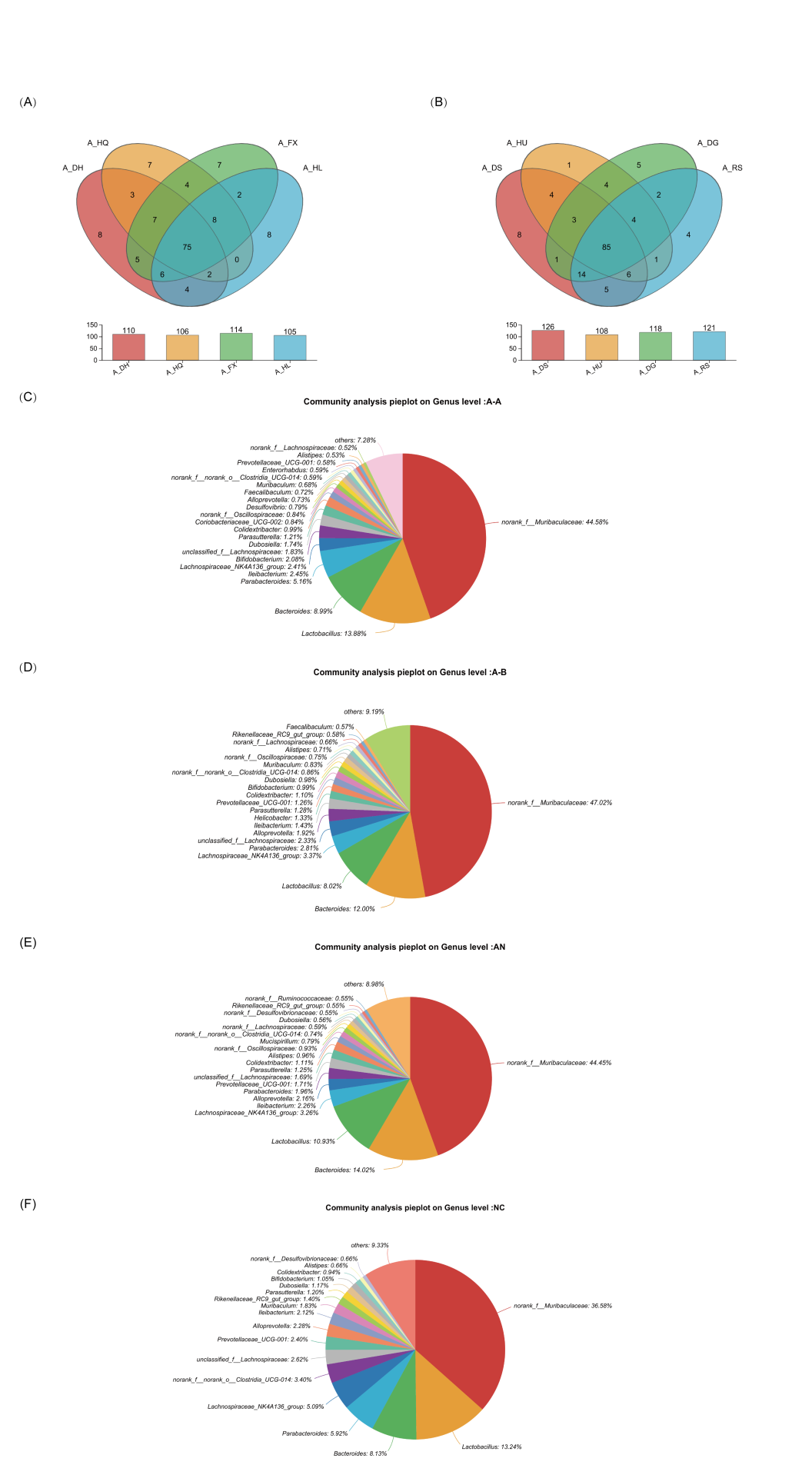


**Figures S12** The effects of traditional Chinese medicines on mouse gut microbiota after 20 days of intervention are presented in the context of dysbiosis. (A) Venn diagram of genus-level composition in the cold-classified drug group. (B) Venn diagram of genus-level composition in the hot-classified drug group. (C) Gut microflora structure in the cold-classified drug group. (D) Gut microflora structure in the hot-classified drug group. (E) Gut microbiota structure in the AN group. (F) Gut microbiota structure in the NC group.
